# Risk of Acute Kidney Injury Associated With Nephrotoxic Burden in Hospitalized Patients: A Scoping Review

**DOI:** 10.1002/cpt.70169

**Published:** 2025-12-21

**Authors:** Wafa Alatawi, Jessica L. Wallace, Dhakrit Rungkitwattanakul, Britney A. Stottlemyer, Tiffany L. Tran, Melanie Manis Reida, Sandra L. Kane‐Gill

**Affiliations:** ^1^ Department of Pharmacy and Therapeutics, School of Pharmacy University of Pittsburgh Pittsburgh Pennsylvania USA; ^2^ Department of Pharmacy Practice, School of Pharmacy University of Tabuk Tabuk Saudi Arabia; ^3^ Department of Pharmacy and Pharmaceutical Sciences Lipscomb University College of Pharmacy Nashville Tennessee USA; ^4^ Department of Clinical and Administrative Pharmacy Science Howard University College of Pharmacy Washington DC USA; ^5^ Division of Nephrology, Department of Medicine University of Alabama at Birmingham Birmingham Alabama USA; ^6^ Department of Pharmacy Practice Samford University Birmingham Alabama USA; ^7^ Department of Pharmacy UPMC Presbyterian Pittsburgh Pennsylvania USA; ^8^ Department of Critical Care Medicine, Program for Critical Care Nephrology University of Pittsburgh School of Medicine Pittsburgh Pennsylvania USA

## Abstract

Limited evidence exists synthesizing the risk of acute kidney injury (AKI) associated with the concomitant administration of multiple nephrotoxic drugs, and even less examining the concept of nephrotoxic burden. The objective of this scoping review was to (1) identify definitions of nephrotoxic burden; (2) methods used to quantify (use of calculations) nephrotoxic burden; and (3) determine the association between nephrotoxic burden and AKI risk. Additionally, we assessed studies reporting the risk of AKI with the concurrent use of three or more nephrotoxic drugs. Following PRISMA guidelines, a comprehensive literature search was conducted. Observational studies in hospitalized patients were included if they assessed nephrotoxic burden or the risk of AKI with concurrent nephrotoxic drug use. Sixteen studies met the inclusion criteria. Four studies assessed nephrotoxic burden, two of which defined and quantified it, and two additional studies adopted those definitions to evaluate associations with AKI. All four reported a significant relationship between increased nephrotoxic burden and AKI risk. Twelve studies evaluated the likelihood of AKI with concurrent administration of three or more nephrotoxic drugs, with reported odds ratios ranging from 1.15 to 3.18 per additional drug. The deleterious effects of concomitant exposure to three or more nephrotoxins on the kidney are evident, stressing a need to take conscious action from a clinician and institutional perspective in the attempt to prevent AKI. Future research should incorporate drug‐specific weighting and consistent reporting standards to improve nephrotoxic burden assessment and guide clinical decision‐making to reduce AKI.

**Study Highlights**

**WHAT IS THE CURRENT KNOWLEDGE ON THE TOPIC?**

Combination therapy with nephrotoxic drugs increases the likelihood of acute kidney injury (AKI) in hospitalized patients. However, there is a lack of studies that specifically assess the cumulative nephrotoxic burden and its association with AKI.

**WHAT QUESTION DID THIS STUDY ADDRESS?**

This scoping review highlights existing evidence on the definition of nephrotoxic burden and its association with AKI. It also identifies and evaluates studies that assess the risk of AKI associated with the concurrent administration of three or more nephrotoxic drugs.

**WHAT DOES THIS STUDY ADD TO OUR KNOWLEDGE?**

This review highlights the observed association between each additional nephrotoxic drug administered and the increased cumulative burden, which consequently increases the likelihood of AKI. It also highlights the limited number of studies that have attempted to measure nephrotoxic burden.

**HOW MIGHT THIS CHANGE CLINICAL PHARMACOLOGY OR TRANSLATIONAL SCIENCE?**

Close monitoring and careful assessment of nephrotoxic burden in clinical practice may lower the incidence of AKI and mitigate its adverse outcomes in hospitalized patients.

Acute kidney injury (AKI) is a significant global health concern, affecting approximately 20% of hospitalized patients, with the incidence rate exceeding 50% among those admitted to intensive care units (ICUs).^1^, ^2^ Medications are a major cause of AKI, with nephrotoxic drugs contributing to 20%–30% of AKI cases in hospitalized patients.^3^, ^4^, ^5^ Drug‐associated AKI (D‐AKI) is associated with a 25% increase in the hospital mortality rate, similar to other AKI etiologies.^6^ Given the role of nephrotoxic medications in AKI development, understanding the impact of combination drug use on the risk of AKI is critical.

The concept of *nephrotoxic burden* has emerged to describe the cumulative nephrotoxic effects of multiple drugs when used concurrently. It is defined as “the cumulative or aggregate exposure to nephrotoxins, considering the nephrotoxic potential of each drug, evaluated at a given time or within a reasonable timeframe depending on the drug’s half‐life in the body.”^7^ This definition suggests that nephrotoxic burden increases with each additional nephrotoxic drug administered or with prolonged exposure to nephrotoxic agents. It also emphasizes the importance of accounting for the nephrotoxic potential of individual drugs, as some agents may cause more severe kidney injury than others.

We aimed to conduct a scoping review to explore broader research questions about nephrotoxic burden. We systematically examined the literature to (1) identify definitions of nephrotoxic burden; (2) methods used to quantify (use of calculations) nephrotoxic burden; and (3) determine the association with the use of calculated nephrotoxic burden and AKI risk. Additionally, we explored studies that reported the likelihood of AKI following the concurrent administration of three or more nephrotoxic agents (no burden calculation used) in hospital settings.

## METHODS

### Search strategy

This scoping review was conducted following the JBI methodology for scoping reviews and in line with the Preferred Reporting Items for Systematic Reviews and Meta‐Analyses extension for Scoping Reviews (PRISMA‐ScR).^8^ The review protocol was developed a priori and is available upon request from the corresponding author. A comprehensive search was developed and subsequently performed in PubMed (NLM), Embase (Ovid), Scopus (Elsevier), and Web of Science (Clarivate Analytics). Semantic Scholar, an artificial intelligence‐based search engine, was also used to identify relevant research. The complete search strategy for all databases is available in **Supplemental Material**. The search strategy aimed to identify published studies without restrictions on the publication date. The reference lists of the most relevant studies were screened for additional literature, referred to as snowballing. Searches for ongoing or unpublished studies were also conducted, including registered clinical trials (ClinicalTrials.gov), registered systematic reviews (PROSPERO), and registered scoping reviews (Open Science Framework (OSF)). The final searches were completed in September 2024, and all results were uploaded to Covidence (Veritas Health Information, Melbourne, Australia) for deduplication.

### Eligibility criteria

Studies examining the association between the effect of drug combination and AKI in hospitalized patients, including those in critical care settings, were included. The inclusion criteria consisted of observational studies, including cohort, case‐control, and cross‐sectional study designs, that explored the association between AKI risk and the use of three or more nephrotoxic drugs, as well as studies that attempted to quantify nephrotoxin burden. The exclusion criteria included non‐English papers, animal studies, review articles, case reports, and case series. No restrictions were placed on age or sex. Studies involving patients with chronic kidney disease or those on dialysis were excluded.

### Evidence selection and screening

All retrieved citations were imported into EndNote v.x20 (Clarivate Analytics, PA, USA), and subsequently uploaded to Covidence, where duplicates were removed. Titles and abstracts were then screened by two independent reviewers to assess eligibility based on the inclusion criteria. Full‐text screening was conducted for studies that met the title and abstract screening criteria, with reasons for exclusion recorded for studies that did not meet the inclusion criteria. Any disagreements between reviewers at any stage of the selection process were resolved through discussion.

### Data extraction

Final included studies were divided for data extraction by two teams; each team had three independent reviewers. Data extracted included the study design, target population, and sample size, as well as whether nephrotoxic burden was assessed as primary or secondary objectives, or as part of additional analysis. We also recorded whether the study provided a clear definition of drug burden or drug combination, how these terms were defined, and the quantitative measures used to assess nephrotoxin burden. Additionally, the number of drugs evaluated, specifying drug names or classes, and the main findings related to AKI risk associated with drug burden or combination therapy were extracted. Any disagreements between reviewers were resolved through discussion. We aimed to map operational definitions of nephrotoxic burden and summarize reported associations with AKI, and did not seek to compile or adjudicate an exhaustive list of all potentially nephrotoxic or renally eliminated medications. In fact, lists such as this have already been reported in the literature.^9^, ^10^ We accepted the drug lists as described by each primary study (e.g., institutional formularies, collaborative lists such as the Nephrotoxic Injury Negated by Just‐in‐time Action (NINJA), or previously published lists) and extracted how nephrotoxic burden was operationalized.

### Outcome

The outcome of interest was AKI in hospitalized patients. Data collected for this outcome included the likelihood of AKI associated with nephrotoxic drug exposure, reported as odds ratios or relative risk. Studies were included if they defined AKI based on established clinical criteria, including the Kidney Disease Improving Global Outcomes (KDIGO) criteria, the modified Risk, Injury, Failure, Loss, End‐stage Kidney Disease (RIFLE) criteria, the pediatric RIFLE criteria, or the Acute Kidney Injury Network (AKIN) criteria.

### Quality assessments

Risk of bias and study quality were assessed using the National Heart, Lung, and Blood Institute (NHLBI) Study Quality Assessment Tools for observational studies, including cohort, case‐control, cross‐sectional, and pre‐post studies. Quality assessment was conducted independently by two teams, with any disagreements resolved through discussion. The NHLBI tool evaluates the internal validity of a study by assessing the extent to which the reported results can be attributed to the exposure being studied rather than flaws in study design or execution. The assessment focuses on key sources of bias, including selection bias, information bias, measurement bias, and confounding. Studies with a high risk of bias are more likely to receive a lower quality rating. The overall study quality was categorized as “good,” “fair,” or “poor” based on these assessments. A study was rated as “good” if it demonstrated a low risk of bias and strong methodological rigor. Studies classified as “fair” had some risk of bias but still provided valid findings, while those rated as “poor” exhibited significant bias, limiting their reliability.

## RESULTS

### Literature search and selection process

**Figure 1:**
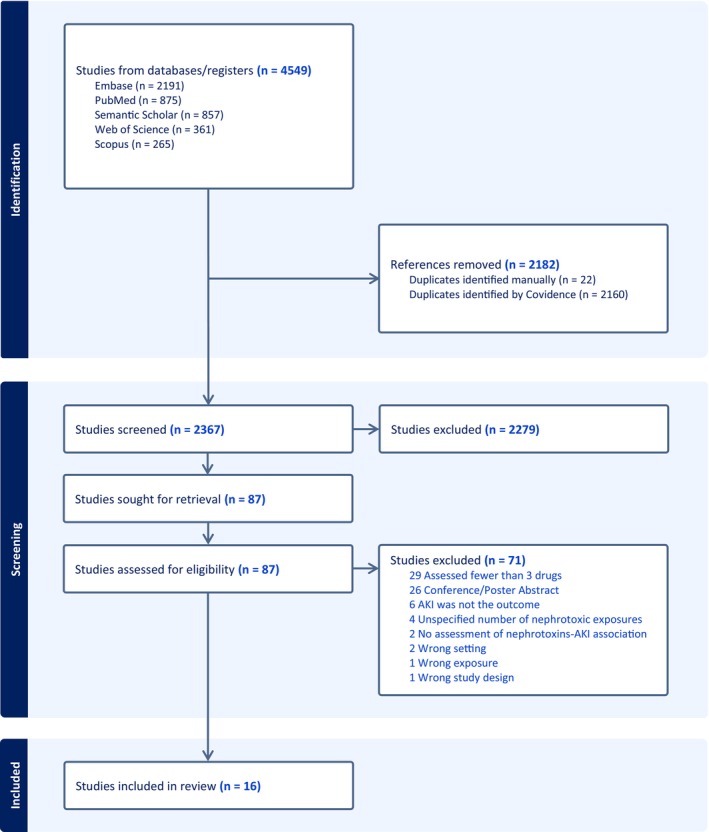
PRISMA flow diagram for article screening and selection. Flowchart illustrating the number of studies identified, screened, included, and excluded during the review process, along with reasons for full‐text exclusion. Sixteen studies were included in the final analysis.

The search identified a total of 4549 studies. Of these, 2182 duplicates were removed, resulting in 2367 references. After screening titles and abstracts, 2279 references were excluded due to irrelevance, leaving 87 papers for full‐text review. Ultimately, 16 papers were included in the final analysis, and 71 papers were excluded for various reasons, as shown in the PRISMA flow diagram (**Figure** **1**).

### Study characteristics

All 16 studies included in this review were retrospective observational studies. Nine studies followed a cohort study design, while one study used a case‐control design, and two studies were nested case‐control studies. Additionally, three studies employed a cross‐sectional design, and one study was quasi‐experimental. Approximately half of the included studies focused on adult patients hospitalized in general wards or critical care units, while the remaining studies examined pediatric patients in similar settings. A subset of studies specifically assessed the risk of AKI associated with concomitant nephrotoxin exposure in distinct patient populations, including adults with cystic fibrosis, children with nephrotic syndrome, pediatric patients who underwent congenital heart surgery, adults diagnosed with carbapenem‐resistant Gram‐negative bacilli receiving high‐dose polymyxin therapy, and very low birth weight infants in the neonatal intensive care unit (NICU). The publication dates of the included studies ranged from 2011 to 2024.

### Nephrotoxic burden definition and quantification approach

Two studies have attempted to quantify nephrotoxic burden and assess its association with the risk of AKI. The first study, a retrospective case‐control study, examined the additive or multiplicative effects of nephrotoxic drugs on AKI risk in hospitalized children. The authors introduced the term “medication exposure intensity” to describe this effect, defining it as “the number of concomitant medication exposures for a study subject.”^11^ For example, if a patient receives three nephrotoxic drugs concurrently, their medication exposure intensity is three. However, if the same three nephrotoxic drugs are administered sequentially without overlapping, the medication exposure intensity remains one.

The second study adopted a similar approach to quantify nephrotoxic burden using a semi‐quantitative method called “nephrotoxic drug‐days.”^12^ In this method, patients receive a nephrotoxic burden score of 1 for each day they are administered a single nephrotoxic drug. If multiple nephrotoxic drugs are administered on the same day, the score corresponds to the total number of nephrotoxic drugs received on that specific day (**Table** **1**).

**Table 1:** Definitions, criteria for multiple drug exposure, drugs evaluated

Source	Nephrotoxic burden/Multiple drug exposure criteria	Drug classes or drug names that were recorded as nephrotoxic agents
Ehrmann (2019)^12^	Nephrotoxic burden was defined in terms of “nephrotoxic drug days.” This approach allowed for a semi‐quantitative evaluation of the overall burden of kidney toxicity experienced by a patient. Essentially, each day a patient took a nephrotoxic drug was counted as one point of nephrotoxic burden. If a patient received multiple nephrotoxic drugs on a particular day, they were assigned a corresponding number of points equivalent to the number of nephrotoxic drugs taken that day	Diuretics (loop diuretics, thiazide diuretics, potassium‐sparing diuretics); antibiotics (vancomycin, aminoglycosides, high‐dose beta‐lactams, sulfamethoxazole‐trimethoprim, rifampicin); antiviral agents (acyclovir); antifungal agents (amphotericin B, voriconazole); renin‐angiotensin‐aldosterone antagonists; hydroxyethyl starch; mannitol; non‐steroidal anti‐inflammatory drugs (including acetylsalicylic acid); immunosuppressants and chemotherapy; iodinated contrast media
Moffett (2011)^11^	“Medication exposure” referred to the count of distinct medications administered to the subjects during their inpatient stay “Medication‐exposure intensity” was defined as the count of concurrent medication exposures for a subject. For instance, if a subject received three medications at the same time, their intensity would be 3. In contrast, a subject who received three medications one after the other, without any overlap, would have an intensity of 1	Acyclovir; amikacin; amphotericin B; cefotaxime; ceftazidime; cisplatin. Colestimethate; ganciclovir; gentamicin; ifosfamide; ketorolac; methotrexate; naproxen; piperacillin with/without tazobactam; vancomycin; ibuprofen; enalapril; cyclosporine; ticarcillin/clavulanate; tobramycin; trimethoprim/sulfamethoxazole
Rheault (2015)^13^	Defined according to Moffett’s definition^11^	Antibiotics (vancomycin, piperacillin/tazobactam, ceftazidime, cefotaxime, cefuroxime, gentamicin, tobramycin, amikacin, other (dapsone, nafcillin)); any calcineurin inhibitor (cyclosporin, tacrolimus); Angiotensin Converting Enzyme Inhibitor (ACEI); Angiotensin Receptor Blockers (ARBs); antiviral (acyclovir); Non‐Steroidal Anti‐Inflammatory Drugs NSAIDs (ibuprofen, ketorolac); other (topiramate, sirolimus)
Chan (2024)^14^	Defined according to Moffett’s definition^11^	This list was based on the hospital formulary and on Moffett’s paper^11^ Acyclovir; adefovir; amikacin; amiloride; amphotericin B; aspirin; captopril; carboplatin; cidofovir; cisplatin; co‐trimoxazole; cyclophosphamide; cyclosporin A; cytarabine; diclofenac sodium; enalapril/enalaprilat; eplerenone; etoposide; everolimus; foscarnet; furosemide; ganciclovir; gemcitabine; gentamicin; hydrochlorothiazide; ibuprofen; ifosfamide; indomethacin; ketorolac; lisinopril; losartan; melphalan; methotrexate; metolazone; naproxen; nivolumab; piperacillin/tazobactam; ramipril; sacubitril and valsartan; sirolimus; spironolactone; tacrolimus; telmisartan; tenofovir; valganciclovir; vancomycin; zoledronic acid
Aysert‐Yildiz (2024)^25^	Definitions of the drugs of interest (polymyxin B and colistimethate sodium (CMS)), including doses and frequencies, were provided. However, it was only stated that concurrent nephrotoxic agents were recorded from patients’ records	Aminoglycosides; diuretics; vasopressors; vancomycin; amphotericin B; ACEI/ARBs; NSAID; contrast agents
Griffin (2023)^21^	This paper follows NINJA to define high nephrotoxic exposure. This is described as the receipt of three or more nephrotoxic medications within a single day, or the administration of intravenous aminoglycoside or vancomycin for a duration of three or more days	The included and assessed nephrotoxins were based on a list of medications in the NINJA algorithm^38^, ^39^ ACEI/ARBs (captopril; enalapril; enalaprilat; lisinopril; losartan; valsartan); antibiotics (amikacin; amphotericin B; clavulanic acid; colistimethate; gentamicin; nafcillin; piperacillin/tazobactam; polymixin B; ticarcillin; tobramycin); antivirals (acyclovir; cidofovir; foscarnet; ganciclovir; tenofovir; valacyclovir; valganciclovir); Calcineurin Inhibitor/Mammalian Target of Rapamycin CI/MTOR‐I (cyclosporine; sirolimus; tacrolimus); iodinated contrast dye (diatrizoate meglumine; diatrizoate sodium; iodixanol; iohexol; iopromide; ioversol; ioxaglate meglumine; ioxilan); NSAIDs (celecoxib; ibuprofen; indomethacin; ketorolac; mesalamine; naproxen); others (carboplatin; cisplatin; deferasirox; ifosfamide; lithium; methotrexate; mitomycin; pamidronate disodium; pentamidine; sulfasalazine; topiramate; zoledronic acid; zonisamide)
Lipp (2022)^28^	A definition of the drugs of interest, including dosing and frequency, was provided. However, concomitant exposure to other nephrotoxins was defined as the administration of any nephrotoxic agents during treatment with the drugs of interest	ACEI; contrast dye; loop diuretics; NSAIDs; sulfa antibiotics; vancomycin; vasopressors; piperacillin/tazobactam; aztreonam; cefepime; ceftazidime; meropenem; ciprofloxacin; levofloxacin
Kim (2022)^24^	It was only mentioned that nephrotoxic agents administered during vancomycin therapy were recorded	Aceclofenac; acetaminophen; acyclovir; albumin; allopurinol; amikacin; amoxicillin; amphotericin B; arbekacin; aspirin; azacitidine; azilsartan; benazepril; bevacizumab; bumetanide; candesartan; captopril; carboplatin; cefaclor; cefadroxil; cefazolin; cefbuperazone; cefdinir; cefditoren; cefepime; cefixime; cefotaxime; cefpodoxime; cefprozil; ceftaroline; ceftazidime; ceftibuten; ceftriaxone; cefuroxime; celecoxib; cephalexine; cidofovir; cisplatin; clindamycin; colistin; contrast media; cyclosporine; dapsone; dexlansoprazol; diclofenac; diflunisal; dobutamine; dopamine; enalapril; entecavir; epinephrine; eprosartan; esomeprazole; ethacrynic acid; etodolac; everolimus; fenoprofen; fluoxetine; flurbiprofen; foscarnet; fosinopril; furosemide; ganciclovir; gemcitabine; gentamicin; hetastarch; hydroxyethylstarch; ibuprofen; ifosfamide; imipenem; immunoglobulin; indinavir; indomethacin; irbesartan; isoproterenol; kanamycin; ketoprofen; ketorolac; lansoprazole; lisinopril; lithium; losartan; mannitol; meclofenamate; mefenamic acid; meloxicam; mesalamine; methotrexate; methylephedrine; milrinone; mitomycin C; moexipril; nabumetone; nafcillin; naproxen; neomycin; norepinephrine; olmesartan; oxaprozin; pantoprazole; paracetamol; perindopril; phenylephrine; piperacillin/tazobactam; piroxicam; polymyxin B; propacetamol; quinapril; ramipril; rifampicin; rifampin; ritonavir; salsalate; sirolimus; streptomycin; streptozocin; sulfadiazine; sulfasalazine; sulindac; tacrolimus; teicoplanin; telmisartan; tenofovir; tobramycin; tolmetin; torsemide; tramadol; trandolapril; trimethoprim‐sulfamethoxazole; valacyclovir; valganciclovir; valsartan; vancomycin; vasopressin; zaltoprofen; zonisamide
Cai (2020)^26^	A clear definition of high‐dose polymyxin B was provided as a dose of at least 30,000 IU/kg/day for ≥ 72 hours during treatment. It was only mentioned that data on concurrent nephrotoxic agent use were collected	Diuretics; vancomycin; aminoglycosides; intravenous contrast; polymyxin B
Hsu (2019)^22^	Concurrent nephrotoxins were defined as the use of any nephrotoxic agents administered 72 hours before, during, or up to 72 hours after the completion of vancomycin therapy	The nephrotoxins’ list included and assessed in this study was based on a previously published list^15^ Acyclovir; amikacin; amphotericin B; captopril; cefotaxime; ceftazidime; cefuroxime; enalapril; enalaprilat; foscarnet; gadopentatdimeglumine; gadoxetate disodium; ganciclovir; gentamicin; ibuprofen; iohexol/iodixanol; indomethacin; ketorolac; nafcillin; piperacillin/tazobactam; ticarcillin/clavulonic acid; topiramate; tobramycin; valacyclovir; valganciclovir; vancomycin
Sridharan (2019)^18^	It was only noted that potentially nephrotoxic drugs administered during vancomycin therapy were documented	Vancomycin; gentamicin; piperacillin/tazobactam; acyclovir; fluconazole; amikacin; fruosemide; cefepime; meropenem; paracetamol; cefotaxime; captopril; ceftriaxone; ganciclovir; ibuprofen; clindamycin; mannitol; acetazolamide; ceftazidime; clarithromycin; metronidazole; co‐trimoxazole; cloxacillin; ampicillin; ciprofloxacin; caspofungin; tigecycline; azithromycin; voriconazole; ganciclovir; levofloxacin; erythromycin; cefuroxime
Jeon (2019)^27^	“Number of nephrotoxic drugs” was defined as the total number of nephrotoxic medications administered on a risk day All drugs had to be administered within 24 hours of each other	101 medications were included and assessed based on four studies^15^, ^40^, ^41^, ^42^; Acyclovir; albumin; allopurinol; amikacin^a^; amphotericin B cholesteryl sulfate complex; amphotericin B deoxylate^a^; amphotericin B lipid complex; amphotericin B liposomal; aspirin; azacitidine; azilsartan; benazepril; bevacizumab; bumetanide; candesartan; captopril; carboplatin; cefaclor; cefadroxil; cefazolin; cefdinir; cefditoren; cefepime; cefixime; cefotaxime; cefpodoxime; cefprozil; ceftaroline; ceftazidime; ceftibuten; ceftriaxone; cefuroxime; celecoxib; cephalexine; cidofovir^a^; cisplatin^a^; clindamycin; colistin^a^; cyclosporine; dapsone; diclofenac; diflunisal; enalapril; eprosartan; ethacrynic acid; etodolac; fenoprofen; fluoxetine; flurbiprofen; foscarnet^a^; fosinopril; furosemide; ganciclovir; gemcitabine; gentamicin^a^; hetastarch; ibuprofen; ifosfamide; imipenem+cilastatin; indinavir; indomethacin; irbesartan; kanamycin^a^; ketoprofen; ketorolac; lisinopril; lithium; losartan; mannitol; meclofenamate; mefenamic acid; meloxicam; mesalamine; methotrexate^a^; mitomycin Ca; moexipril; nabumetone; nafcillin; naproxen; neomycin^a^; Olmesartan; oxaprozin; paromomycin; perindopril; piperacillin; piroxicam; polymyxin B sulfate^a^; quinapril; ramipril; rifampin; ritonavir; salsalate; sirolimus; streptomycin^a^; streptozocin^a^; sulfadiazine; sulfasalazine; sulindac; tacrolimus^a^; telmisartan; tenofovir^a^; tobramycin^a^; tolmetin; torsemide; trandolapril; trimethoprim‐sulfamethoxazole; valacyclovir; valganciclovir; valsartan; vancomycin^a^; zonisamide
Uber (2018)^17^	Nephrotoxin exposures were defined according to the Nephrotoxic Injury Negated by Just‐in‐time Action (NINJA) collaborative; high exposure was defined as receipt of ≥ 3 nephrotoxins concurrently or three consecutive days of intravenous aminoglycoside	The list of nephrotoxins assessed in this paper was derived from previous papers^15^, ^16^, ^38^ Acyclovir; amikacin; amphotericin B; aspirin; captopril; carboplatin; cefotaxime; ceftazidime; cefuroxime; celecoxib; cidofovir; cisplatin; colistimethate; contrast; cyclosporine; dapsone; enalapril; enalaprilat; foscarnet; ganciclovir; gentamicin; ibuprofen; ifosfamide; indomethacin; ketorolac; lisinopril; lithium; losartan; mesalamine; methotrexate; mitomycin; nafcillin; naproxen; pamidronate disodium; pentamidine; piperacillin; piperacillin/tazobactam; sirolimus; sulfasalazine; tacrolimus; tenofovir; ticarcillin/clavulanic acid; tobramycin; topiramate; valacyclovir; valganciclovir; valsartan; vancomycin; zolendronic acid; zonisamide
Soares (2018)^20^	It was only stated that data were collected on the number of medications prescribed per patient	Fentanyl; propofol; morphine; dobutamine; omeprazole; amikacin; norepinephrine; amiodarone; magnesium sulfate; carvedilol; furosemide; spironolactone; vancomycin; amoxicillin/clavulanic acid; diazepam; ciprofloxacin; simvastatin; valproic acid; metronidazole; clopidogrel; ceftriaxone; non‐ionic contrast media; meropenem; hydralazine; polymyxin; clarithromycin; clonazepam; clonidine; phenytoin; gentamicin; captopril; calcium gluconate; amlodipine; phenobarbital; risperidone; clindamycin; losartan; epinephrine; sodium nitroprusside; hydrochlorothiazide; acetaminophen; oxacillin; piperacillin/tazobactam; atropine; tramadol; cefepime; cefazolin
Barhight (2017)^19^	A definition for concurrent nephrotoxin combinations was not provided. However, nephrotoxic doses separated by more than 24 hours from the preceding dose were considered separate exposures	The list of nephrotoxic drugs evaluated in this study was based on a published paper^15^ Acyclovir; amikacin; amphotericin B; captopril; cefotaxime; ceftazidime; cefuroxime; enalapril; enalaprilat; foscarnet; gadopentatdimeglumine; gadoxetate disodium; ganciclovir; gentamicin; ibuprofen; iohexol/iodixanol; indomethacin; ketorolac; nafcillin; piperacillin/tazobactam; ticarcillin/lavulonic acid; topiramate; tobramycin; valcyclovir; valganciclovir; vancomycin
Karino (2016)^23^	Combination therapy with vancomycin and piperacillin‐tazobactam was defined as the initiation of both drugs within 24 hours of one another and co‐administration for at least 48 hours. However, no definition was provided for concomitant nephrotoxic agents	Vasopressors; aminoglycosides; colistin; angiotensin‐converting enzyme inhibitors; angiotensin II receptor blockers; diuretics; intravenous contrast

^a^ High‐risk nephrotoxic medication.

### Association of AKI and nephrotoxic burden using burden calculations

The risk of AKI development or progression has been linked to an increased nephrotoxic burden. A matched case‐control study conducted in an adult ICU setting found that a higher nephrotoxic burden was significantly associated with a 20% increased likelihood of AKI development or worsening with each additional drug, ((OR) of 1.20 (95% CI: 1.04–1.38, *P* = 0.015)), compared to controls (0.86 ± 1.30).^12^ Similarly, findings by Moffett and Goldstein indicated that among non‐critically ill pediatric patients, each increase in medication exposure intensity was associated with a 1.7‐fold higher odds of AKI development or worsening (95% CI: 1.04–2.9, *P* = 0.03). The study also reported that the proportion of patients developing AKI increased as nephrotoxic drug exposure intensity increased.^11^ Two additional studies applied the nephrotoxic medication exposure intensity definition established by Moffett and Goldstein to examine its association with AKI. One study, which assessed nephrotoxic exposure intensity in hospitalized children, found that each unit increase in nephrotoxic medication exposure intensity was associated with a 34% higher odds of AKI (OR: 1.34, 95% CI: 1.09–1.65, *P* = 0.01).^13^ Another study evaluated the nephrotoxic exposure intensity among patients who were admitted to the pediatric ICU. This study reported that the relative risk of AKI associated with medication exposure intensity was 1.38 times higher (95% CI: 1.10–1.73, *P* = 0.005). Furthermore, this risk was significantly elevated in patients receiving four or more nephrotoxic medications concurrently, with a relative risk of 3.69 (95% CI: 1.32–10.30, *P* = 0.01)^14^ (**Table** **2**).

**Table 2:** Association of AKI and nephrotoxic burden

Author (year)	Study design	Population and sample size	Assessing nephrotoxin burden: Primary, secondary objectives, or additional analysis?	Nephrotoxic drugs burden is clearly defined	Number of drugs evaluated for the association with AKI	Findings: association between nephrotoxic burden/or concomitant drugs and AKI	Interpretation	Overall quality assessment
Ehrmann (2019)^12^	Nested case‐control study	ICU adult patients *N* = 1001	Primary objective	Yes	Mean (SD) of nephrotoxic burden was 0.86 ± 1.30 drug days among controls and 1.20 ± 1.76 drug days among cases	OR 1.20 (95% CI: 1.04–1.38; *P* = 0.015)	For each unit increase in nephrotoxic burden, the odds of developing or worsening AKI increased by 20%	Good
Moffett (2011)^11^	Retrospective case‐control	Non‐ICU pediatric patients *N* = 714	Secondary objective	Yes	Median nephrotoxic medication exposure intensity was 2 (range, 0–7)	OR 1.7 (95% CI: 1.0–2.9; *P* = 0.03)	Patients who developed AKI had 70% higher odds of having been exposed to at least one nephrotoxic medication compared to those who did not develop AKI	Good
Rheault (2015)^13^	Retrospective cohort study	Non‐ICU pediatric patients *N* = 336 children	Secondary objective	Yes	The mean (SD) number of medication exposure intensity in the AKI group was 0.92 ± 1.03	OR 1.34 (95% CI: 1.09–1.65; *P* = 0.01)	Each unit increase in medication exposure intensity increases the odds of AKI by 34%	Fair
Chan (2025)^14^	Retrospective cohort study	Pediatric ICU patients *N* = 253	Primary objective	Yes	Among children with AKI, the medication exposure intensity ranged from 0 to 6	Relative risk (RR) of nephrotoxic medication exposure intensity is 1.381 (95% CI: 1.101–1.732) RR of using four or more nephrotoxic medications is 3.687 (95% CI: 1.320–10.301)	For each 1 unit increase in nephrotoxic medication exposure intensity increases the relative risk of AKI by 38.1% Patients using four or more nephrotoxic medications had a 268.7% higher risk of developing AKI than those with fewer nephrotoxic exposures	Good

### Association between AKI and exposure to three or more nephrotoxic drugs (no calculation needed)

Four studies examined the risk of AKI associated with the concurrent use of three or more nephrotoxic drugs in ICU patients. One study assessed nephrotoxic drug use among children admitted to the cardiovascular ICU following congenital heart surgery. This study defined nephrotoxin exposure according to the Nephrotoxic Injury Negated by Just‐in‐time Action (NINJA) collaborative, where high exposure was identified as either the concurrent use of three or more nephrotoxic medications or intravenous aminoglycoside administration for three consecutive days.^15^, ^16^ However, nephrotoxin exposure did not have a statistically significant association with AKI development; the adjusted relative risk was 1.2 (95% CI: 0.8–1.8).^17^ Another study evaluated the likelihood of AKI development among children admitted to the ICU who received vancomycin during their stay. Findings indicated that the odds of AKI increased by 1.7 (95% CI: 1.1–2.5) for each additional concomitant nephrotoxic drug used during vancomycin therapy.^18^ Similarly, a study conducted among very low birth weight infants in the neonatal ICU found that the odds of AKI increased by 83% for each additional nephrotoxic drug added to the patients’ regimen (OR 1.83; 95% CI: 1.33–2.53, *P* = 0.0002).^19^ Lastly, one study examined this association in adult ICU patients and found no statistically significant difference in the number of concurrently used nephrotoxic drugs between patients who developed AKI and those who did not. However, when assessing AKI in relation to the total number of prescribed drugs, regardless of nephrotoxicity, a significant association was observed (OR 1.15; 95% CI: 1.05–1.26)^20^ (**Tables** **1** **and** **3**).

**Table 3:** Association of AKI and the concomitant use of three or more nephrotoxins

Source	Study design	Population and sample size	AKI association with concomitant use of ≥ 3 nephrotoxins assessed as primary, secondary, or additional analysis?	The concomitant use of nephrotoxic drugs is clearly defined	Number of drugs evaluated for burden	Findings; association between nephrotoxin burden/or concomitant drugs and AKI	Interpretation	Overall quality assessment
Aysert‐Yildiz (2024)^25^	Retrospective cohort study	Hospitalized adult patients *N* = 290	Secondary objective	No	Three	AOR 2.45 (95% CI: 1.29–4.65, *P* = 0.006)	Patients who were treated with polymyxin and at least two additional nephrotoxic medications had 2.45 times higher odds of developing AKI compared to those who did not	Fair
Griffin (2023)^21^	Retrospective cohort study	Non‐ICU Adult patients *N* = 2532	Primary objective	Yes	Three nephrotoxins or more	OR 1.82 (95% CI: 1.50–2.22, *P* < 0.001)	Patients exposed to three nephrotoxic drugs or more had 82% higher odds of developing AKI compared to unexposed patients	Fair
Lipp (2022)^28^	Retrospective cohort study	Non‐ICU adult cystic fibrosis patients *N* = 156	Additional analysis	No	1 or more doses of nephrotoxins during colistimethate sodium or tobramycin therapy	AOR 2.51 (95% CI: 1.27–4.95)	Among patients receiving either intravenous colistimethate sodium or tobramycin, those who were also given concomitant nephrotoxic medications had 2.5 times higher odds of developing AKI, compared to those who did not receive additional nephrotoxins	Fair
Kim (2022)^24^	Retrospective observational study	Hospitalized adult patients *N* = 104	Primary objective	No	Six nephrotoxins or more	AOR 3.18 (95% CI: 1.14–8.88)	Patients who received six or more nephrotoxic medications had over three times the odds of developing AKI compared to those who received fewer nephrotoxins	Poor
Cai (2020)^26^	Retrospective cohort study	Hospitalized adult patients *N* = 43	Additional analysis	No	Median number of concurrent nephrotoxins in addition to polymyxin = 3 (IQR 2–3)	AOR 2.14, (95% CI: 1.03–4.45, *P* = 0.04)	Patients with a higher number of concurrent nephrotoxins during polymyxin B therapy had a higher odds of developing AKI	Fair
Hsu (2019)^22^	Quasi‐experimental study	Hospitalized children *N* = 386	Primary objective	Yes	Three nephrotoxins or more	AOR 1.40 (95% CI: 1.06–1.85, *P* = 0.019)	For each additional nephrotoxin, there was a 40% increase in the odds of AKI	Fair
Sridharan (2019)^18^	Retrospective observational	ICU Pediatric patients *N* = 102	Additional analysis	No	The median number (range) of concomitant drugs with potential nephrotoxicity was three^1^, ^2^, ^3^, ^4^, ^5^, ^6^, ^7^, ^8^	OR 1.7 (95% CI: 1.1–2.5)	The odds of AKI increased by 70% with increasing in the number of concomitants drugs with nephrotoxic potential	Fair
Jeon (2019)^27^	Retrospective cohort study	Hospitalized adult patients *N* = 62,561	Primary objective	Yes	Mean (SD) number of nephrotoxic medications per hospital day = 1.56 ± 0.89	OR 1.40 (95% CI: 1.16–1.70, *P* < 0.05)	The odds of developing AKI increased by 40% for each additional highly nephrotoxic drug administered	Good
Uber (2018)^17^	Retrospective cohort	Cardiovascular ICU pediatric patients *N* = 154	Primary objective	Yes	Three nephrotoxins or more	ARR 1.2 (95% CI: 0.8–1.8)	Nephrotoxin exposure was not statistically significant and associated with the development of AKI	Good
Soares (2018)^20^	Cross‐sectional	Adult ICU patients *N* = 122	Primary objective	No	Mean (SD) of potential nephrotoxins was 9.3 (4.6)	OR 1.15 (95% CI: 1.05–1.26)	Non‐significant difference between the number of potentially nephrotoxic drugs used in non‐AKI vs. AKI pts (*P* = 0.06). However, the total number of drugs was significantly associated with AKI	Fair
Barhight (2017)^19^	Retrospective cohort study	Very low birth weight infants in NICU *N* = 233	Secondary objective	No	Median number of nephrotoxin exposure among AKI patients was 5 (IRQ 3–9)	OR 1.83 (95% CI: 1.33–2.53, *P* = 0.0002)	For each additional nephrotoxic medication, the odds of developing AKI increased by 83%	Fair
Karino (2016)^23^	Nested‐case control	Hospitalized adult patients *N* = 320 patients	Secondary objective	No	Three	OR 2.33 (95% CI, 1.34–4.04)	Patients who received any concomitant nephrotoxic medication along with an antibiotic combination therapy had 2.33 times higher odds of developing AKI compared to those who did not receive additional nephrotoxins	Fair

Eight studies examined the association between AKI and the concurrent use of nephrotoxic drugs in non‐critically ill patients. One study focused on adult patients hospitalized for at least 48 hours, defining nephrotoxic exposure according to the NINJA collaborative. Nephrotoxic drug exposure was associated with higher odds of developing AKI (OR 1.82; 95% CI: 1.50–2.22, *P* < 0.001).^21^ Three studies assessed the likelihood of AKI with concurrent nephrotoxic drug use among patients receiving vancomycin therapy. One study defined concurrent nephrotoxic drugs as the use of any nephrotoxic agents 72 hours before, during, or up to 72 hours after vancomycin therapy. They found that for each additional nephrotoxic drug, the odds of AKI increased by 40%, with an adjusted odds ratio of 1.40 (95% CI: 1.06–1.85, *P* = 0.019).^22^ Another study reported that the odds of AKI in adult patients who received any concomitant nephrotoxin during antibiotic combination therapy were 2.33 (95% CI: 1.34–4.04).^23^ Lastly, one study found that during vancomycin therapy, patients receiving six or more nephrotoxins had an adjusted odds ratio of 3.18 (95% CI: 1.14–8.88) for AKI^24^ (**Tables** **1** **and** **3**).

Two studies evaluated the odds of AKI development among hospitalized adults receiving polymyxin therapy with concurrent nephrotoxic drug use. The adjusted odds of AKI ranged between 2.14 and 2.4 (95% CI: 1.03–4.45; *P* = 0.04, 95% CI: 1.29–4.65; *P* = 0.006) among patients who were administered at least two concomitant nephrotoxic drugs in addition to polymyxin.^25^, ^26^ Another study developed a dynamic model to predict AKI among adult patients during the first 5 days of hospitalization. The highest odds of developing AKI associated with nephrotoxic drugs were observed on day four of hospitalization, OR 1.40 (95% CI: 1.16–1.70, *P* value < 0.05). These findings suggest that for each additional nephrotoxic drug, the odds of AKI increased by 40%.^27^ Lastly, a study assessed the odds of AKI development among hospitalized adults diagnosed with cystic fibrosis who received treatment with either colistimethate sodium or tobramycin along with concurrent nephrotoxic drugs. The adjusted odds of AKI in these patients were 2.51 (95% CI: 1.27–4.95) for each additional dose of nephrotoxic drug administered during the treatment course with either antibiotics^28^ (**Tables** **1** **and** **3**).

### Quality assessment

Of the 16 studies included in this review, five were rated as having “good” overall quality, indicating low risk of bias and sound methodological rigor. Ten studies were rated as “fair,” suggesting moderate risk of bias due to some limitations, but their findings were still considered valid. One study was rated as “poor” due to several methodological issues, including a high risk of selection bias from excluding many patients due to incomplete records, failure to adjust for potential confounders, and a lack of clarity in defining the exposure. These quality assessments reflect the variability in methodological robustness across the included studies and should be considered when interpreting the strength and reliability of the reported findings.

## DISCUSSION

This scoping review examined studies measuring nephrotoxic burden and associated risk of AKI, uncovering a major gap in the literature. Strikingly, only two studies and one review have attempted to define and quantify nephrotoxic burden while assessing its association with AKI, highlighting the need for further research in this crucial area.^7^, ^11^, ^12^ Both studies defined nephrotoxic burden by calculating the number of nephrotoxic drugs administered concurrently on the same day, assigning one point for each drug. This method indicates that nephrotoxic burden increases with each additional nephrotoxic drug given simultaneously. Despite minor differences in the terminology, both studies employed a similar and straightforward approach to define nephrotoxic burden. However, neither study incorporated a weighted schema to account for variations in nephrotoxic potential among different drugs, as suggested by others.^7^ Two additional studies adopted the method proposed by Moffett and Goldstein.^13^, ^14^ The findings across all four studies indicated that an increased nephrotoxic burden or intensity was associated with the development or worsening of AKI, with the strength of this association increasing as the burden increased.

This method of measuring nephrotoxic burden is readily applicable at the patient’s bedside. However, it does not account for the varying degrees of nephrotoxicity associated with different drugs. Ehrmann *et al*. and Moffett *et al*. quantified nephrotoxic burden using a predefined list of nephrotoxic medications derived either from literature reviews or their institutional formulary. However, they did not assign weights to drugs based on their relative nephrotoxic potential. Similarly, the drug lists used in the remaining included studies were either based on institutional formularies, adapted from the list published by Moffett and Goldstein, or adopted from the nephrotoxic list developed by the NINJA collaborative initiative.^15^ More recently, Gray *et al*. employed a modified Delphi method to achieve expert consensus on nephrotoxic drugs relevant to adult ICU populations. Out of 167 candidate medications, 20 were identified as having probable or probable/definite nephrotoxicity in ICU settings.^29^ Further, Stottlemyer *et al*. also used expert consensus to assess the weighting of nephrotoxic drugs in non‐ICU patients, identifying 10 drugs with probable or probable/definite nephrotoxicity.^30^ Although the use of a standardized list helps clinicians assess the potential risk of AKI associated with these drugs, incorporating a weighted approach that reflects the relative nephrotoxic potential of each drug could enhance the precision of nephrotoxic burden assessments and better inform clinical decision‐making.^7^ At present, there is no standardized or validated method for assigning weights to drugs according to their relative nephrotoxic potential. Possible weighting approaches could include association with AKI frequency and the mechanism of damage contributed to the drug.

Across studies, the definition of “nephrotoxin” reflects the primary studies’ lists, which vary by setting. This review did not attempt to comprehensively screen all potentially nephrotoxic medications. As a result, some heterogeneity in exposure classification is expected. However, recent initiatives have compiled broader, standardized lists of potentially nephrotoxic or renally eliminated drugs. For example, Barreto *et al*. developed a comprehensive list of 681 agents, offering a uniform reference point for defining nephrotoxic exposure.^9^ Such standardized lists can improve comparability across studies and reduce misclassification by providing consistent exposure definitions. Beyond harmonization, an additional step forward would be to categorize drugs by their underlying mechanisms of nephrotoxicity, for instance, hemodynamic effects (ACE inhibitors), direct tubular toxicity (aminoglycosides), tubular obstruction (high‐dose methotrexate), or immune‐mediated injury (acute interstitial nephritis).^31^ Notably, some lists also flag agents that cause pseudo‐AKI—drugs that elevate serum creatinine by inhibiting tubular creatinine secretion—which can produce an apparent decline in estimated Glomerular Filtration Rate (eGFR) without true loss of filtration.^32^ This further emphasizes the need to include mechanisms in studied lists of nephrotoxic drugs.^31^ In general, drugs causing only pseudo‐AKI would not be included as part of a burden assessment. Overall, the mechanistic classification can help serve as a more structured form of “weighting,” enriching both clinical interpretation and future research frameworks for assessing nephrotoxic burden.

We also examined studies evaluating the association between AKI and the administration of three or more nephrotoxic medications. Findings indicate that the odds of AKI development or worsening increase by a range of 1.15–3.18 times with each additional nephrotoxic drug. Similar trends have been observed in studies assessing the risk of AKI with two nephrotoxic drugs compared to one, or one nephrotoxic drug compared to no exposure. For instance, a study assessing neonatal nephrotoxic exposure and its association with AKI found that the adjusted hazard ratio (aHR) for AKI at any stage among neonates who received both aminoglycoside and another nephrotoxic medication was 2.68 (95% CI: 1.35–5.33). In contrast, the aHR for neonates who received aminoglycoside alone was 1.92 (95% CI: 1.16–3.19). These findings indicate that the hazard of developing AKI increases when an additional nephrotoxic drug is administered.^33^ Another study examined the odds of AKI among ICU patients exposed to different nephrotoxic medications, reporting an increased risk among patients receiving vancomycin (OR 1.3; 95% CI 1.0–1.6), tacrolimus (OR 2.1; 95% CI 1.2–3.6), and gentamicin (OR 1.8; 95% CI 1.4–2.4).^34^ Additionally, increased risk of AKI has been associated with the “triple whammy”—the concurrent administration of diuretics, nonsteroidal anti‐inflammatory drugs, and either angiotensin‐converting enzyme inhibitors or angiotensin receptor antagonists—with an adjusted rate ratio of 1.31 (95% CI: 1.12–1.53).^35^ The variability in effect estimates across studies may reflect differences in the number and types of nephrotoxic drugs evaluated, the degree of adjustment for confounding variables, and the specific nephrotoxic potential of the medications involved. These studies did not use one of the proposed calculations for nephrotoxic burden, so duration of exposure was not a consideration. Still, two studies out of the 16 that we identified did not support an association. However, these studies contained small sample sizes and were conducted in ICUs, where residual confounders could be a factor.^17^, ^20^ To better elucidate the impact of cumulative nephrotoxic burden, well‐designed interventional or pharmacoepidemiological studies are needed.

Future prospective, interventional studies will be required to determine whether strategies designed to reduce nephrotoxic burden can effectively lower AKI incidence. However, observational quality‐improvement initiatives have already provided encouraging evidence. For example, the NINJA program, initially piloted at a single center and later expanded to nine pediatric hospitals, demonstrated sustained reductions in nephrotoxic medication exposure and a 23.8% decrease in nephrotoxic medication–associated AKI rates. The program relied on systematic screening for high nephrotoxin exposure (≥ 3 drugs on the same day or ≥ 3 days of aminoglycosides) and implemented daily creatinine monitoring.^36^ These results underscore the potential of interventional strategies, though rigorous prospective studies in broader patient populations will be necessary to confirm causality and generalizability.

Few studies included in this scoping review provided a clear definition of concurrent nephrotoxic drug administration, specifically regarding dosage, administration windows, and duration of therapy or nephrotoxin potential. While some studies acknowledged collecting data on concurrent nephrotoxic drug use, they lacked detailed information on how these drugs were administered concurrently. There was no consideration of evaluation of concomitant use within a reasonable time frame, depending on the half‐life of the drug in the body, considering a drug may be discontinued but still have a pharmacologic effect.^7^ This has been suggested as part of the definition for nephrotoxic burden.^7^ Moreover, there are inconsistencies in definitions, methodologies, and nephrotoxins assessed. The absence of a standardized framework for assessing nephrotoxic burden, along with variability in concurrent nephrotoxic drug administration reporting, underscores the need for further research. Future studies should focus on refining nephrotoxic burden measurement by incorporating drug‐specific nephrotoxicity weighting and standardizing reporting criteria for nephrotoxic drug combinations, clarifying the timing/window of concomitant use. Establishing these measures may enhance clinical decision‐making, inform nephrotoxic stewardship programs, and ultimately reduce the incidence and severity of AKI in hospitalized patients.

A nephrotoxin stewardship program is a promising approach to standardize the identification of nephrotoxic and renally eliminated drugs and to guide prescribing and monitoring.^7^ Such programs are coordinated patient‐care strategies aimed at medication safety, kidney health, and cost avoidance. Core elements typically include (i) standardized, routinely updated lists of nephrotoxins/renally cleared drugs; (ii) automated clinical decision support to flag high‐risk exposure (e.g., multiple concurrent nephrotoxins or prolonged aminoglycoside/vancomycin use); (iii) pharmacist‐nephrology review for substitution/deprescribing and dose optimization; (iv) therapeutic drug monitoring, where appropriate; and (v) proactive kidney monitoring using functional and damage/stress biomarkers. Collectively, these elements operationalize real‐time risk mitigation and align prescribing with kidney‐safety principles across inpatient settings.^7^

Most included studies adjusted for confounding factors in their analyses based on the data available. However, confounding by indication remains a key limitation when interpreting nephrotoxic burden metrics. Patients prescribed multiple nephrotoxic drugs may represent those with greater severity of illness, which itself increases the risk of AKI. For example, one study conducted a cause‐specific analysis to address this issue and found that the adjusted association between nephrotoxic exposure and AKI was weaker than the crude estimate, suggesting that part of the observed risk may reflect underlying illness rather than drug exposure alone.^37^ While statistical adjustments help reduce bias, residual confounding by indication is likely unavoidable in observational studies. Prospective or interventional studies are therefore needed to clarify whether reducing nephrotoxic burden truly decreases the incidence of AKI.

To our knowledge, this is the first review to systematically examine nephrotoxic burden and the risk of AKI associated with the concurrent administration of at least three or more nephrotoxic drugs. However, this review has limitations. For the objective of determining the association between AKI and nephrotoxin exposure (no calculation needed), this was often not the primary objective of the study, so our search could have overlooked other studies that did not have this as the primary objective, since the results could not have been apparent in a title and abstract search. Still, our comprehensive search strategy incorporated five databases, including Semantic Scholar, an AI‐based search engine designed to identify connections and hidden relationships between topics. Furthermore, while we critically appraised the included studies in alignment with our objectives, some studies did not explicitly assess the association between AKI and three nephrotoxic drugs as their primary focus; consequently, certain quality criteria were not applicable when evaluating their analysis of this association. This would lean toward interpreting studies as low quality. Three or more drugs were the emphasis of nephrotoxic burden because data support the risk for AKI at this point,^11^ certainly two nephrotoxins or other risks, such as drug exposure (duration and dose), are considerations beyond the scope of this review. Most nephrotoxic drug effects are dose dependent, and when possible, understanding dose and drug concentrations as part of causal evaluation, but unfortunately, this information was not reported in the studies included in this scoping review. Moreover, all included evidence is observational; therefore, risks of selection bias, information bias, and residual confounding‐ including confounding by indication—cannot be excluded. While the use of consensus AKI criteria (KDIGO/AKIN/RIFLE) improves comparability, heterogeneity remains substantial across patient groups (e.g., children vs. adults), care settings (ICU vs. non‐ICU), and severity of illness. These differences hamper interpretation and limit cross‐study comparability, and they contributed to our decision to present a narrative synthesis rather than a pooled estimate.

## CONCLUSION

This scoping review clarifies how nephrotoxic burden is defined and measured across the literature, indicating that only a few studies have focused on defining and measuring nephrotoxic burden and assessing its association with AKI. The deleterious effects of concomitant exposure to three or more nephrotoxins on the kidney are highlighted, stressing the need to take conscious action from a clinician and institutional perspective in an attempt to prevent AKI. Still, there is an evidence gap that remains to understand nephrotoxic burden management through the reduction of burden or a weighted burden or duration of overlap in nephrotoxin exposure to improve patient kidney outcomes.

## FUNDING

No funding was received for this work.

## CONFLICT OF INTEREST

Dr. Kane‐Gill receives grant funding from the National Institute of Diabetes and Digestive and Kidney Diseases R01 DK121730 and U01DK130010, the National Center for Complementary and Integrative Health U54AT008909, and the Jewish Healthcare Foundation. All other authors declared no competing interests in this work.

## AUTHOR CONTRIBUTIONS

W.A., J.L.W., D.R., B.A.S., and S.L.K.‐G. wrote the manuscript. S.L.K.‐G. designed the research. W.A., J.L.W., D.R., B.A.S., T.L.T., M.M.R., and S.L.K.‐G. performed the research. W.A. and J.L.W. analyzed the data.

## Supporting information


Data S1.
