# Supplementary material for: Risk of Acute Kidney Injury Associated With Nephrotoxic Burden in Hospitalized Patients: A Scoping Review
Source: Clin Pharmacol Ther. 2025 Dec 21;119(4):891–904. doi: 10.1002/cpt.70169 (PMC12997509; doi:10.1002/cpt.70169)
Supplement: Supplementary file 1 — Data S1. [file CPT-119-891-s001.docx]

**Supplemental Material 1: Search strategy for all databases**

The final searches were completed in September 2024. No date limit used.

**PubMed – limited to English language; 415 results; 459 results 9.2.2024**

("acute kidney injury"[MeSH] OR "acute kidney injur*"[tiab] OR "acute renal injur*"[tiab] OR "acute renal insufficienc*"[tiab] OR "acute kidney insufficienc*"[tiab] OR "acute kidney failure*"[tiab] OR "acute renal failure*"[tiab] OR "acute kidney damage*"[tiab] OR "acute renal damage*"[tiab]) AND (nephrotoxi*[tiab] OR "nephro toxi*"[tiab] OR "kidney toxi*"[tiab] OR "renal toxi*"[tiab]) AND ("Drug Therapy, Combination"[Mesh] OR "drug polytherap*"[tiab] OR "combination drug therap*"[tiab] OR "Drug Combinations"[MeSH] OR "drug combination*"[tiab] OR "combination therap*"[tiab] OR "drug combination therap*"[tiab] OR "medication combination*"[tiab] OR "combination medic*"[tiab] OR "combined medic*"[tiab] OR "combination drug regimen*"[tiab] OR "combined drug regimen*"[tiab] OR "multi-drug regimen*"[tiab] OR "multidrug regimen*"[tiab] OR "multiple drug regimen*"[tiab] OR "combination drug treatment*"[tiab] OR "combination pharmacotherap*"[tiab] OR "combined pharmacotherap*"[tiab] OR "combined drug therap*"[tiab] OR "combined drug treatment*"[tiab] OR "concomitant drug therap*"[tiab] OR "concomitant drug*"[tiab] OR "concomitant medication*"[tiab] OR "drug burden*"[tiab] OR "drug exposure*"[tiab] OR "medication exposure*"[tiab] OR "drug intensit*"[tiab] OR "Prescription Drugs"[MeSH] OR "prescription drug*"[tiab] OR "Amlodipine Besylate, Olmesartan Medoxomil Drug Combination"[MeSH] OR alea[tiab] OR amelior[tiab] OR "Amlodipine Besylate Olmesartan Medoxomil"[tiab] OR "amlodipine plus Olmesartan"[tiab] OR "amlodipine/Olmesartan"[tiab] OR Azor[tiab] OR balzak[tiab] OR bivis[tiab] OR capenon[tiab] OR forzaten[tiab] OR "olmesartan medoxomil plus amlodipine"[tiab] OR "olmesartan medoxomil/amlodipine besylate"[tiab] OR "olmesartan plus amlodipine"[tiab] OR "olmesartan/amlodipine"[tiab] OR sevikar[tiab] OR vocado[tiab] OR "Amlodipine, Valsartan Drug Combination"[MeSH] OR "amlodipine besylate/valsartan"[tiab] OR "amlodipine plus valsartan"[tiab] OR "Amlodipine Valsartan"[tiab] OR copalia[tiab] OR dafiro[tiab] OR Exforge[tiab] OR imprida[tiab] OR "valsartan plus amlodipine"[tiab] OR "valsartan/amlodipine"[tiab] OR "Amoxicillin-Potassium Clavulanate Combination"[MeSH] OR aclam[tiab] OR aktil[tiab] OR amocla[tiab] OR amoclan[tiab] OR amoclav[tiab] OR amoksiklav[tiab] OR "Amox clav"[tiab] OR "Amoxi Clavulanate"[tiab] OR "amoxicillin and clavulanate potassium"[tiab] OR "Amoxicillin Clavulanic Acid"[tiab] OR "amoxicillin plus clavulanate potassium"[tiab] OR "amoxicillin plus clavulanic acid"[tiab] OR "Amoxicillin Potassium Clavulanate"[tiab] OR "amoxicillin/clavulanate potassium"[tiab] OR amoxiclav[tiab] OR "Amoxycillin Clavulanic Acid"[tiab] OR ancla[tiab] OR augmentan[tiab] OR Augmentin[tiab] OR augmentine[tiab] OR bioclavid[tiab] OR "BRL 25000"[tiab] OR BRL25000[tiab] OR cavumox[tiab] OR ciblor[tiab] OR clamax[tiab] OR clamoxin[tiab] OR clavam[tiab] OR clavamox[tiab] OR clavubactin[tiab] OR clavucid[tiab] OR "clavulanate potassium/amoxicillin"[tiab] OR "Clavulanate Potentiated Amoxycillin"[tiab] OR "clavulanic acid/amoxicillin"[tiab] OR Clavulin[tiab] OR "Co amoxiclav"[tiab] OR "co amoxyclav"[tiab] OR Coamoxiclav[tiab] OR coamoxyclav[tiab] OR curam[tiab] OR duomox[tiab] OR enhancin[tiab] OR fleming[tiab] OR forcid[tiab] OR "forcid solutab"[tiab] OR moxiclav[tiab] OR spektramox[tiab] OR stacillin[tiab] OR strenzen[tiab] OR Synulox[tiab] OR taromentin[tiab] OR xiclav[tiab] OR "Cilastatin, Imipenem Drug Combination"[MeSH] OR "cilastatin plus imipenem"[tiab] OR "cilastatin sodium/imipenem"[tiab] OR "Cilastatin, Imipenem"[tiab] OR "Imipenem Cilastatin"[tiab] OR "imipenem plus cilastatin"[tiab] OR "MK 0787 MK 0791 mixture"[tiab] OR "MK 787 MK 791 mixture"[tiab] OR prepenem[tiab] OR Primaxin[tiab] OR tenacid[tiab] OR Thienam[tiab] OR tienam[tiab] OR tienem[tiab] OR Zienam[tiab] OR "Efavirenz, Emtricitabine, Tenofovir Disoproxil Fumarate Drug Combination"[MeSH] OR Atripla[tiab] OR "efavirenz/emtricitabine/tenofovir disoproxil"[tiab] OR "efavirenz/tenofovir disoproxil fumarate/emtricitabine"[tiab] OR "emtricitabine/tenofovir disoproxil fumarate/efavirenz"[tiab] OR "emtricitabine/tenofovir disoproxil/efavirenz"[tiab] OR "tenofovir disoproxil fumarate/emtricitabine/efavirenz"[tiab] OR "tenofovir disoproxil/emtricitabine/efavirenz"[tiab] OR "Elvitegravir, Cobicistat, Emtricitabine, Tenofovir Disoproxil Fumarate Drug Combination"[MeSH] OR "Elvitegravir Cobicistat Emtricitabine Tenofovir Disoproxil Fumarate"[tiab] OR "Elvitegravir, Cobicistat, Emtricitabine, and Tenofovir Disoproxil Fumarate"[tiab] OR "elvitegravir/cobicistat/emtricitabine/tenofovir df"[tiab] OR "elvitegravir/cobicistat/emtricitabine/tenofovir disoproxil fumarate"[tiab] OR Genvoya[tiab] OR "Quad Pill"[tiab] OR stribild[tiab] OR "Emtricitabine, Rilpivirine, Tenofovir Drug Combination"[MeSH] OR Complera[tiab] OR "Emtricitabine Rilpivirine Tenofovir"[tiab] OR "emtricitabine/rilpivirine/tenofovir disoproxil fumarate"[tiab] OR "emtricitabine/tenofovir disoproxil fumarate/rilpivirine"[tiab] OR "emtricitabine/tenofovir disoproxil/rilpivirine"[tiab] OR eviplera[tiab] OR "rilpivirine/emtricitabine/tenofovir disoproxil"[tiab] OR "rilpivirine/tenofovir disoproxil fumarate/emtricitabine"[tiab] OR "tenofovir disoproxil fumarate/emtricitabine/rilpivirine"[tiab] OR "tenofovir disoproxil/emtricitabine/rilpivirine"[tiab] OR "Emtricitabine, Tenofovir Disoproxil Fumarate Drug Combination"[MeSH] OR "emtricitabine plus tenofovir disoproxil"[tiab] OR "Emtricitabine Tenofovir Disoproxil Fumarate"[tiab] OR "emtricitabine/tenofovir disoproxil"[tiab] OR "emtriva-viread"[tiab] OR "tenofovir disoproxil fumarate plus emtricitabine"[tiab] OR "tenofovir disoproxil fumarate/emtricitabine"[tiab] OR "tenofovir disoproxil plus emtricitabine"[tiab] OR "tenofovir disoproxil/emtricitabine"[tiab] OR Torad[tiab] OR Truvada[tiab] OR "Piperacillin, Tazobactam Drug Combination"[MeSH] OR "piperacillin and tazobactam"[tiab] OR "piperacillin plus tazobactam"[tiab] OR "piperacillin sodium plus tazobactam"[tiab] OR "Piperacillin Tazobactam"[tiab] OR "Pipercillin Sodium Tazobactam Sodium"[tiab] OR tazip[tiab] OR "tazobactam plus piperacillin"[tiab] OR "Tazobactam, Piperacillin"[tiab] OR Tazocel[tiab] OR Tazocillin[tiab] OR tazocilline[tiab] OR Tazocin[tiab] OR tazonam[tiab] OR tazorex[tiab] OR "yp 14"[tiab] OR "yp 18"[tiab] OR yp14[tiab] OR yp18[tiab] OR zobactam[tiab] OR Zosyn[tiab] OR "Sitagliptin Phosphate, Metformin Hydrochloride Drug Combination"[MeSH] OR efficib[tiab] OR Janumet[tiab] OR "metformin plus sitagliptin"[tiab] OR "metformin-sitagliptin"[tiab] OR "mk 0431a"[tiab] OR mk0431a[tiab] OR ristfor[tiab] OR "Sitagliptin Phosphate Metformin Hydrochloride"[tiab] OR "sitagliptin plus metformin"[tiab] OR "sitagliptin-metformin"[tiab] OR velmetia[tiab] OR "Tobramycin, Dexamethasone Drug Combination"[MeSH] OR "dexamethasone plus tobramycin"[tiab] OR "dexamethasone-tobramycin"[tiab] OR TobraDex[tiab] OR "tobramycin and dexamethasone"[tiab] OR "tobramycin-dexamethasone"[tiab] OR "Trimethoprim, Sulfamethoxazole Drug Combination"[MeSH] OR Abactrim[tiab] OR alfatrim[tiab] OR "apo sulfatrim"[tiab] OR bactar[tiab] OR Bactifor[tiab] OR bactramin[tiab] OR Bactrim[tiab] OR bactrimel[tiab] OR bethaprim[tiab] OR Biseptol[tiab] OR Biseptol480[tiab] OR Centran[tiab] OR Centrin[tiab] OR chemotrim[tiab] OR "Co Trimoxazole"[tiab] OR comox[tiab] OR cotrim[tiab] OR Cotrimoxazole[tiab] OR "co-trimoxazole"[tiab] OR deprim[tiab] OR Drylin[tiab] OR duratrimet[tiab] OR eltrianyl[tiab] OR escoprim[tiab] OR Eslectin[tiab] OR espectrin[tiab] OR Eusaprim[tiab] OR fectrim[tiab] OR groprim[tiab] OR helveprim[tiab] OR imexim[tiab] OR Kepinol[tiab] OR lagatrim[tiab] OR Lescot[tiab] OR linaris[tiab] OR microtrim[tiab] OR nopil[tiab] OR oecotrim[tiab] OR omsat[tiab] OR Oriprim[tiab] OR potesept[tiab] OR resprim[tiab] OR "ro 6 2580-11"[tiab] OR "ro 62580"[tiab] OR Septra[tiab] OR septran[tiab] OR septrim[tiab] OR Septrin[tiab] OR septrine[tiab] OR sigaprim[tiab] OR soltrim[tiab] OR sulfamethoprim[tiab] OR "sulfamethoprim-ds"[tiab] OR "sulfamethoxazole and trimethoprim"[tiab] OR "sulfamethoxazole plus trimethoprim"[tiab] OR "sulfamethoxazole trimethoprim"[tiab] OR sulfaprim[tiab] OR sulfatrim[tiab] OR "sulfatrim-ds"[tiab] OR "sulfatrim-ss"[tiab] OR sulfotrim[tiab] OR sulmeprim[tiab] OR Sulprim[tiab] OR sumetrolim[tiab] OR sumetrolin[tiab] OR supracombin[tiab] OR thiocuran[tiab] OR "TMP SMX"[tiab] OR "tms forte"[tiab] OR trib[tiab] OR trigonyl[tiab] OR Trimedin[tiab] OR "trimeth/sulfa"[tiab] OR "trimethoprim plus sulfamethoxazole"[tiab] OR "Trimethoprim Sulfamethoxazole"[tiab] OR Trimethoprimsulfa[tiab] OR trimethoprimsulfamethoxazole[tiab] OR "trimethoprim-sulfamethoxazole"[tiab] OR "trimetoprim-sulfa"[tiab] OR trimetoprimsulfamethoxazole[tiab] OR trimezol[tiab] OR trimforte[tiab] OR Trimosulfa[tiab] OR trimoxazole[tiab] OR "uro ts d"[tiab] OR "uroplus ds"[tiab] OR "uroplus ss"[tiab])

**Embase (Ovid) – limited to English language; 1015 results**

| No. | Query | Results |
| --- | --- | --- |
| #306 | #10 AND #16 AND #305 AND [english]/lim | 1015 |
| #305 | #17 OR #18 OR #19 OR #20 OR #21 OR #22 OR #23 OR #24 OR #25 OR #26 OR #27 OR #28 OR #29 OR #30 OR #31 OR #32 OR #33 OR #34 OR #35 OR #36 OR #37 OR #38 OR #39 OR #40 OR #41 OR #42 OR #43 OR #44 OR #45 OR #46 OR #47 OR #48 OR #49 OR #50 OR #51 OR #52 OR #53 OR #54 OR #55 OR #56 OR #57 OR #58 OR #59 OR #60 OR #61 OR #62 OR #63 OR #64 OR #65 OR #66 OR #67 OR #68 OR #69 OR #70 OR #71 OR #72 OR #73 OR #74 OR #75 OR #76 OR #77 OR #78 OR #79 OR #80 OR #81 OR #82 OR #83 OR #84 OR #85 OR #86 OR #87 OR #88 OR #89 OR #90 OR #91 OR #92 OR #93 OR #94 OR #95 OR #96 OR #97 OR #98 OR #99 OR #100 OR #101 OR #102 OR #103 OR #104 OR #105 OR #106 OR #107 OR #108 OR #109 OR #110 OR #111 OR #112 OR #113 OR #114 OR #115 OR #116 OR #117 OR #118 OR #119 OR #120 OR #121 OR #122 OR #123 OR #124 OR #125 OR #126 OR #127 OR #128 OR #129 OR #130 OR #131 OR #132 OR #133 OR #134 OR #135 OR #136 OR #137 OR #138 OR #139 OR #140 OR #141 OR #142 OR #143 OR #144 OR #145 OR #146 OR #147 OR #148 OR #149 OR #150 OR #151 OR #152 OR #153 OR #154 OR #155 OR #156 OR #157 OR #158 OR #159 OR #160 OR #161 OR #162 OR #163 OR #164 OR #165 OR #166 OR #167 OR #168 OR #169 OR #170 OR #171 OR #172 OR #173 OR #174 OR #175 OR #176 OR #177 OR #178 OR #179 OR #180 OR #181 OR #182 OR #183 OR #184 OR #185 OR #186 OR #187 OR #188 OR #189 OR #190 OR #191 OR #192 OR #193 OR #194 OR #195 OR #196 OR #197 OR #198 OR #199 OR #200 OR #201 OR #202 OR #203 OR #204 OR #205 OR #206 OR #207 OR #208 OR #209 OR #210 OR #211 OR #212 OR #213 OR #214 OR #215 OR #216 OR #217 OR #218 OR #219 OR #220 OR #221 OR #222 OR #223 OR #224 OR #225 OR #226 OR #227 OR #228 OR #229 OR #230 OR #231 OR #232 OR #233 OR #234 OR #235 OR #236 OR #237 OR #238 OR #239 OR #240 OR #241 OR #242 OR #243 OR #244 OR #245 OR #246 OR #247 OR #248 OR #249 OR #250 OR #251 OR #252 OR #253 OR #254 OR #255 OR #256 OR #257 OR #258 OR #259 OR #260 OR #261 OR #262 OR #263 OR #264 OR #265 OR #266 OR #267 OR #268 OR #269 OR #270 OR #271 OR #272 OR #273 OR #274 OR #275 OR #276 OR #277 OR #278 OR #279 OR #280 OR #281 OR #282 OR #283 OR #284 OR #285 OR #286 OR #287 OR #288 OR #289 OR #290 OR #291 OR #292 OR #293 OR #294 OR #295 OR #296 OR #297 OR #298 OR #299 OR #300 OR #301 OR #302 OR #303 OR #304 | 732337 |
| #304 | 'uroplus ss':ab,ti | 0 |
| #303 | 'uroplus ds':ab,ti | 0 |
| #302 | 'uro ts d':ab,ti | 1 |
| #301 | 'trimoxazole':ab,ti | 4347 |
| #300 | 'trimosulfa':ab,ti | 0 |
| #299 | 'trimforte':ab,ti | 0 |
| #298 | 'trimezol':ab,ti | 2 |
| #297 | 'trimetoprimsulfamethoxazole':ab,ti | 22 |
| #296 | 'trimetoprim-sulfa':ab,ti | 1 |
| #295 | 'trimethoprim-sulfamethoxazole':ab,ti | 12129 |
| #294 | 'trimethoprimsulfamethoxazole':ab,ti | 7945 |
| #293 | 'trimethoprimsulfa':ab,ti | 53 |
| #292 | 'trimethoprim sulfamethoxazole':ab,ti | 12113 |
| #291 | 'trimethoprim plus sulfamethoxazole':ab,ti | 31 |
| #290 | 'trimeth/sulfa':ab,ti | 4 |
| #289 | 'trimedin':ab,ti | 3 |
| #288 | 'trigonyl':ab,ti | 0 |
| #287 | 'trib':ab,ti | 137 |
| #286 | 'tms forte':ab,ti | 1 |
| #285 | 'tmp smx':ab,ti | 2385 |
| #284 | 'thiocuran':ab,ti | 0 |
| #283 | 'supracombin':ab,ti | 0 |
| #282 | 'sumetrolin':ab,ti | 2 |
| #281 | 'sumetrolim':ab,ti | 23 |
| #280 | 'sulprim':ab,ti | 15 |
| #279 | 'sulmeprim':ab,ti | 0 |
| #278 | 'sulfotrim':ab,ti | 3 |
| #277 | 'sulfatrim-ss':ab,ti | 0 |
| #276 | 'sulfatrim-ds':ab,ti | 0 |
| #275 | 'sulfatrim':ab,ti | 13 |
| #274 | 'sulfaprim':ab,ti | 3 |
| #273 | 'sulfamethoxazole trimethoprim':ab,ti | 2269 |
| #272 | 'sulfamethoxazole plus trimethoprim':ab,ti | 30 |
| #271 | 'sulfamethoxazole and trimethoprim':ab,ti | 469 |
| #270 | 'sulfamethoprim-ds':ab,ti | 0 |
| #269 | 'sulfamethoprim':ab,ti | 2 |
| #268 | 'soltrim':ab,ti | 11 |
| #267 | 'sigaprim':ab,ti | 0 |
| #266 | 'septrine':ab,ti | 2 |
| #265 | 'septrin':ab,ti | 172 |
| #264 | 'septrim':ab,ti | 7 |
| #263 | 'septran':ab,ti | 34 |
| #262 | 'septra':ab,ti | 50 |
| #261 | 'ro 62580':ab,ti | 7 |
| #260 | 'ro 6 2580-11':ab,ti | 1 |
| #259 | 'resprim':ab,ti | 3 |
| #258 | 'potesept':ab,ti | 0 |
| #257 | 'oriprim':ab,ti | 3 |
| #256 | 'omsat':ab,ti | 7 |
| #255 | 'oecotrim':ab,ti | 0 |
| #254 | 'nopil':ab,ti | 2 |
| #253 | 'microtrim':ab,ti | 0 |
| #252 | 'linaris':ab,ti | 1 |
| #251 | 'lescot':ab,ti | 6 |
| #250 | 'lagatrim':ab,ti | 0 |
| #249 | 'kepinol':ab,ti | 2 |
| #248 | 'imexim':ab,ti | 0 |
| #247 | 'helveprim':ab,ti | 0 |
| #246 | 'groprim':ab,ti | 0 |
| #245 | 'fectrim':ab,ti | 0 |
| #244 | 'eusaprim':ab,ti | 35 |
| #243 | 'espectrin':ab,ti | 0 |
| #242 | 'eslectin':ab,ti | 4 |
| #241 | 'escoprim':ab,ti | 0 |
| #240 | 'eltrianyl':ab,ti | 0 |
| #239 | 'duratrimet':ab,ti | 0 |
| #238 | 'drylin':ab,ti | 2 |
| #237 | 'deprim':ab,ti | 6 |
| #236 | 'co-trimoxazole':ab,ti | 4333 |
| #235 | 'cotrimoxazole':ab,ti | 9081 |
| #234 | 'cotrim':ab,ti | 15 |
| #233 | 'comox':ab,ti | 28 |
| #232 | 'co trimoxazole':ab,ti | 4332 |
| #231 | 'chemotrim':ab,ti | 0 |
| #230 | 'centrin':ab,ti | 597 |
| #229 | 'centran':ab,ti | 2 |
| #228 | 'biseptol480':ab,ti | 4 |
| #227 | 'biseptol':ab,ti | 110 |
| #226 | 'bethaprim':ab,ti | 0 |
| #225 | 'bactrimel':ab,ti | 0 |
| #224 | 'bactrim':ab,ti | 986 |
| #223 | 'bactramin':ab,ti | 2 |
| #222 | 'bactifor':ab,ti | 0 |
| #221 | 'bactar':ab,ti | 1 |
| #220 | 'apo sulfatrim':ab,ti | 0 |
| #219 | 'alfatrim':ab,ti | 0 |
| #218 | 'abactrim':ab,ti | 2 |
| #217 | 'cotrimoxazole'/exp | 93422 |
| #216 | 'tobramycin-dexamethasone':ab,ti | 90 |
| #215 | 'tobramycin and dexamethasone':ab,ti | 55 |
| #214 | 'tobradex':ab,ti | 90 |
| #213 | 'dexamethasone-tobramycin':ab,ti | 11 |
| #212 | 'dexamethasone plus tobramycin':ab,ti | 1 |
| #211 | 'dexamethasone plus tobramycin'/exp | 1233 |
| #210 | 'velmetia':ab,ti | 3 |
| #209 | 'sitagliptin-metformin':ab,ti | 137 |
| #208 | 'sitagliptin plus metformin':ab,ti | 24 |
| #207 | 'sitagliptin phosphate metformin hydrochloride':ab,ti | 4 |
| #206 | 'ristfor':ab,ti | 2 |
| #205 | 'mk0431a':ab,ti | 3 |
| #204 | 'mk 0431a':ab,ti | 2 |
| #203 | 'metformin-sitagliptin':ab,ti | 111 |
| #202 | 'metformin plus sitagliptin':ab,ti | 14 |
| #201 | 'janumet':ab,ti | 39 |
| #200 | 'efficib':ab,ti | 2 |
| #199 | 'metformin plus sitagliptin'/exp | 545 |
| #198 | 'zosyn':ab,ti | 324 |
| #197 | 'zobactam':ab,ti | 4 |
| #196 | 'yp18':ab,ti | 0 |
| #195 | 'yp14':ab,ti | 12 |
| #194 | 'yp 18':ab,ti | 8 |
| #193 | 'yp 14':ab,ti | 17 |
| #192 | 'tazorex':ab,ti | 0 |
| #191 | 'tazonam':ab,ti | 1 |
| #190 | 'tazocin':ab,ti | 272 |
| #189 | 'tazocilline':ab,ti | 14 |
| #188 | 'tazocillin':ab,ti | 14 |
| #187 | 'tazocel':ab,ti | 0 |
| #186 | 'tazobactam, piperacillin':ab,ti | 382 |
| #185 | 'tazobactam plus piperacillin':ab,ti | 3 |
| #184 | 'tazip':ab,ti | 1 |
| #183 | 'pipercillin sodium tazobactam sodium':ab,ti | 0 |
| #182 | 'piperacillin tazobactam':ab,ti | 9031 |
| #181 | 'piperacillin sodium plus tazobactam':ab,ti | 2 |
| #180 | 'piperacillin plus tazobactam':ab,ti | 51 |
| #179 | 'piperacillin and tazobactam':ab,ti | 248 |
| #178 | 'piperacillin plus tazobactam'/exp | 37664 |
| #177 | 'truvada':ab,ti | 620 |
| #176 | 'torad':ab,ti | 5 |
| #175 | 'tenofovir disoproxil/emtricitabine':ab,ti | 27 |
| #174 | 'tenofovir disoproxil plus emtricitabine':ab,ti | 1 |
| #173 | 'tenofovir disoproxil fumarate/emtricitabine':ab,ti | 370 |
| #172 | 'tenofovir disoproxil fumarate plus emtricitabine':ab,ti | 18 |
| #171 | 'emtriva-viread':ab,ti | 0 |
| #170 | 'emtricitabine/tenofovir disoproxil':ab,ti | 361 |
| #169 | 'emtricitabine tenofovir disoproxil fumarate':ab,ti | 336 |
| #168 | 'emtricitabine plus tenofovir disoproxil':ab,ti | 10 |
| #167 | 'emtricitabine plus tenofovir disoproxil'/exp | 4718 |
| #166 | 'tenofovir disoproxil/emtricitabine/rilpivirine':ab,ti | 1 |
| #165 | 'tenofovir disoproxil fumarate/emtricitabine/rilpivirine':ab,ti | 4 |
| #164 | 'rilpivirine/tenofovir disoproxil fumarate/emtricitabine':ab,ti | 6 |
| #163 | 'rilpivirine/emtricitabine/tenofovir disoproxil':ab,ti | 24 |
| #162 | 'eviplera':ab,ti | 67 |
| #161 | 'emtricitabine/tenofovir disoproxil/rilpivirine':ab,ti | 2 |
| #160 | 'emtricitabine/tenofovir disoproxil fumarate/rilpivirine':ab,ti | 1 |
| #159 | 'emtricitabine/rilpivirine/tenofovir disoproxil fumarate':ab,ti | 14 |
| #158 | 'emtricitabine rilpivirine tenofovir':ab,ti | 46 |
| #157 | 'complera':ab,ti | 38 |
| #156 | 'emtricitabine plus rilpivirine plus tenofovir disoproxil'/exp | 432 |
| #155 | 'stribild':ab,ti | 109 |
| #154 | 'quad pill':ab,ti | 10 |
| #153 | 'genvoya':ab,ti | 62 |
| #152 | 'elvitegravir/cobicistat/emtricitabine/tenofovir disoproxil fumarate':ab,ti | 58 |
| #151 | 'elvitegravir/cobicistat/emtricitabine/tenofovir df':ab,ti | 61 |
| #150 | 'elvitegravir, cobicistat, emtricitabine, and tenofovir disoproxil fumarate':ab,ti | 18 |
| #149 | 'elvitegravir cobicistat emtricitabine tenofovir disoproxil fumarate':ab,ti | 58 |
| #148 | 'cobicistat plus elvitegravir plus emtricitabine plus tenofovir disoproxil'/exp | 567 |
| #147 | 'tenofovir disoproxil/emtricitabine/efavirenz':ab,ti | 1 |
| #146 | 'tenofovir disoproxil fumarate/emtricitabine/efavirenz':ab,ti | 16 |
| #145 | 'emtricitabine/tenofovir disoproxil/efavirenz':ab,ti | 1 |
| #144 | 'emtricitabine/tenofovir disoproxil fumarate/efavirenz':ab,ti | 1 |
| #143 | 'efavirenz/tenofovir disoproxil fumarate/emtricitabine':ab,ti | 9 |
| #142 | 'efavirenz/emtricitabine/tenofovir disoproxil':ab,ti | 42 |
| #141 | 'atripla':ab,ti | 259 |
| #140 | 'efavirenz plus emtricitabine plus tenofovir disoproxil'/exp | 1671 |
| #139 | 'zienam':ab,ti | 1 |
| #138 | 'tienem':ab,ti | 1 |
| #137 | 'tienam':ab,ti | 80 |
| #136 | 'thienam':ab,ti | 16 |
| #135 | 'tenacid':ab,ti | 2 |
| #134 | 'primaxin':ab,ti | 33 |
| #133 | 'prepenem':ab,ti | 2 |
| #132 | 'mk 787 mk 791 mixture':ab,ti | 0 |
| #131 | 'mk 0787 mk 0791 mixture':ab,ti | 0 |
| #130 | 'imipenem plus cilastatin':ab,ti | 21 |
| #129 | 'imipenem cilastatin':ab,ti | 1993 |
| #128 | 'cilastatin, imipenem':ab,ti | 16 |
| #127 | 'cilastatin sodium/imipenem':ab,ti | 3 |
| #126 | 'cilastatin plus imipenem':ab,ti | 1 |
| #125 | 'cilastatin plus imipenem'/exp | 5596 |
| #124 | 'xiclav':ab,ti | 1 |
| #123 | 'taromentin':ab,ti | 1 |
| #122 | 'synulox':ab,ti | 7 |
| #121 | 'strenzen':ab,ti | 3 |
| #120 | 'stacillin':ab,ti | 1 |
| #119 | 'spektramox':ab,ti | 7 |
| #118 | 'moxiclav':ab,ti | 2 |
| #117 | 'forcid solutab':ab,ti | 1 |
| #116 | 'forcid':ab,ti | 1 |
| #115 | 'fleming':ab,ti | 1669 |
| #114 | 'enhancin':ab,ti | 52 |
| #113 | 'duomox':ab,ti | 7 |
| #112 | 'curam':ab,ti | 6 |
| #111 | 'coamoxyclav':ab,ti | 81 |
| #110 | 'coamoxiclav':ab,ti | 1210 |
| #109 | 'co amoxyclav':ab,ti | 66 |
| #108 | 'co amoxiclav':ab,ti | 1122 |
| #107 | 'clavulin':ab,ti | 12 |
| #106 | 'clavulanic acid/amoxicillin':ab,ti | 202 |
| #105 | 'clavulanate potentiated amoxycillin':ab,ti | 18 |
| #104 | 'clavulanate potassium/amoxicillin':ab,ti | 1 |
| #103 | 'clavucid':ab,ti | 0 |
| #102 | 'clavubactin':ab,ti | 2 |
| #101 | 'clavamox':ab,ti | 15 |
| #100 | 'clavam':ab,ti | 59 |
| #99 | 'clamoxin':ab,ti | 1 |
| #98 | 'clamax':ab,ti | 1 |
| #97 | 'ciblor':ab,ti | 3 |
| #96 | 'cavumox':ab,ti | 1 |
| #95 | 'brl25000':ab,ti | 80 |
| #94 | 'brl 25000':ab,ti | 28 |
| #93 | 'bioclavid':ab,ti | 1 |
| #92 | 'augmentine':ab,ti | 16 |
| #91 | 'augmentin':ab,ti | 1304 |
| #90 | 'augmentan':ab,ti | 16 |
| #89 | 'ancla':ab,ti | 5 |
| #88 | 'amoxycillin clavulanic acid':ab,ti | 519 |
| #87 | 'amoxiclav':ab,ti | 1250 |
| #86 | 'amoxicillin/clavulanate potassium':ab,ti | 107 |
| #85 | 'amoxicillin potassium clavulanate':ab,ti | 34 |
| #84 | 'amoxicillin plus clavulanic acid':ab,ti | 151 |
| #83 | 'amoxicillin plus clavulanate potassium':ab,ti | 1 |
| #82 | 'amoxicillin clavulanic acid':ab,ti | 4403 |
| #81 | 'amoxicillin and clavulanate potassium':ab,ti | 48 |
| #80 | 'amoxi clavulanate':ab,ti | 4 |
| #79 | 'amox clav':ab,ti | 40 |
| #78 | 'amoksiklav':ab,ti | 12 |
| #77 | 'amoclav':ab,ti | 1 |
| #76 | 'amoclan':ab,ti | 1 |
| #75 | 'amocla':ab,ti | 3 |
| #74 | 'aktil':ab,ti | 3 |
| #73 | 'aclam':ab,ti | 32 |
| #72 | 'amoxicillin plus clavulanic acid'/exp | 48317 |
| #71 | 'valsartan/amlodipine':ab,ti | 180 |
| #70 | 'valsartan plus amlodipine':ab,ti | 12 |
| #69 | 'imprida':ab,ti | 1 |
| #68 | 'exforge':ab,ti | 41 |
| #67 | 'dafiro':ab,ti | 1 |
| #66 | 'copalia':ab,ti | 1 |
| #65 | 'amlodipine valsartan':ab,ti | 202 |
| #64 | 'amlodipine plus valsartan':ab,ti | 10 |
| #63 | 'amlodipine besylate/valsartan':ab,ti | 13 |
| #62 | 'amlodipine plus valsartan'/exp | 463 |
| #61 | 'vocado':ab,ti | 1 |
| #60 | 'sevikar':ab,ti | 15 |
| #59 | 'olmesartan/amlodipine':ab,ti | 132 |
| #58 | 'olmesartan plus amlodipine':ab,ti | 6 |
| #57 | 'olmesartan medoxomil/amlodipine besylate':ab,ti | 19 |
| #56 | 'olmesartan medoxomil plus amlodipine':ab,ti | 3 |
| #55 | 'forzaten':ab,ti | 1 |
| #54 | 'capenon':ab,ti | 1 |
| #53 | 'bivis':ab,ti | 3 |
| #52 | 'balzak':ab,ti | 1 |
| #51 | 'azor':ab,ti | 54 |
| #50 | 'amlodipine/olmesartan':ab,ti | 61 |
| #49 | 'amlodipine plus olmesartan':ab,ti | 4 |
| #48 | 'amlodipine besylate olmesartan medoxomil':ab,ti | 11 |
| #47 | 'amelior':ab,ti | 2 |
| #46 | 'alea':ab,ti | 67 |
| #45 | 'amlodipine plus olmesartan'/exp | 260 |
| #44 | 'prescription drug*':ab,ti | 15155 |
| #43 | 'prescription drug'/exp | 12820 |
| #42 | 'drug intensit*':ab,ti | 48 |
| #41 | 'medication exposure*':ab,ti | 1662 |
| #40 | 'drug exposure*':ab,ti | 16573 |
| #39 | 'drug burden*':ab,ti | 495 |
| #38 | 'concomitant medication*':ab,ti | 7439 |
| #37 | 'concomitant drug*':ab,ti | 2352 |
| #36 | 'concomitant drug therap*':ab,ti | 223 |
| #35 | 'combined drug treatment*':ab,ti | 304 |
| #34 | 'combined pharmacotherap*':ab,ti | 307 |
| #33 | 'combined drug therap*':ab,ti | 367 |
| #32 | 'combination drug treatment*':ab,ti | 242 |
| #31 | 'multiple drug regimen*':ab,ti | 279 |
| #30 | 'multidrug regimen*':ab,ti | 887 |
| #29 | 'multi-drug regimen*':ab,ti | 359 |
| #28 | 'combined drug regimen*':ab,ti | 85 |
| #27 | 'combination drug regimen*':ab,ti | 162 |
| #26 | 'combined medic*':ab,ti | 2179 |
| #25 | 'combination medic*':ab,ti | 660 |
| #24 | 'medication combination*':ab,ti | 372 |
| #23 | 'drug combination therap*':ab,ti | 695 |
| #22 | 'combination therap*':ab,ti | 97889 |
| #21 | 'drug polytherap*':ab,ti | 71 |
| #20 | 'drug combination*':ab,ti | 21815 |
| #19 | 'combination drug therap*':ab,ti | 862 |
| #18 | 'drug combination'/exp | 235395 |
| #17 | 'combination drug therapy'/exp | 212431 |
| #16 | #11 OR #12 OR #13 OR #14 OR #15 | 86058 |
| #15 | 'renal toxi*':ab,ti | 7430 |
| #14 | 'kidney toxi*':ab,ti | 1130 |
| #13 | 'nephro toxi*':ab,ti | 163 |
| #12 | 'nephrotoxi*':ab,ti | 37258 |
| #11 | 'nephrotoxicity'/exp | 70815 |
| #10 | #1 OR #2 OR #3 OR #4 OR #5 OR #6 OR #7 OR #8 OR #9 | 140070 |
| #9 | 'acute renal damage*':ab,ti | 248 |
| #8 | 'acute kidney damage*':ab,ti | 229 |
| #7 | 'acute renal failure*':ab,ti | 34294 |
| #6 | 'acute kidney insufficienc*':ab,ti | 139 |
| #5 | 'acute renal insufficienc*':ab,ti | 2122 |
| #4 | 'acute renal injur*':ab,ti | 2046 |
| #3 | 'acute kidney injur*':ab,ti | 58070 |
| #2 | 'acute kidney failur*':ab,ti | 1834 |
| #1 | 'acute kidney failure'/exp | 122257 |

**Embase (Elsevier) – Ovid /exp = Elsevier /de; 1148 results on 9.2.2024**

'combination drug therapy'/de OR 'combination drug therapy’:ti,ab OR 'drug polytherap*':ti,ab OR 'combination drug therap*':ti,ab OR 'drug combination'/de OR 'drug combination’:ti,ab OR 'drug combination*':ti,ab OR 'combination therap*':ti,ab OR 'drug combination therap*':ti,ab OR 'medication combination*':ti,ab OR 'combination medic*':ti,ab OR 'combined medic*':ti,ab OR 'combination drug regimen*':ti,ab OR 'combined drug regimen*':ti,ab OR 'multi-drug regimen*':ti,ab OR 'multidrug regimen*':ti,ab OR 'multiple drug regimen*':ti,ab OR 'combination drug treatment*':ti,ab OR 'combination pharmacotherap*':ti,ab OR 'combined pharmacotherap*':ti,ab OR 'combined drug therap*':ti,ab OR 'combined drug treatment*':ti,ab OR 'concomitant drug therap*':ti,ab OR 'concomitant drug*':ti,ab OR 'concomitant medication*':ti,ab OR 'drug burden*':ti,ab OR 'drug exposure*':ti,ab OR 'medication exposure*':ti,ab OR 'drug intensit*':ti,ab OR 'prescription drug'/de OR 'prescription drug’:ti,ab OR 'prescription drug*':ti,ab OR 'amlodipine plus olmesartan'/de OR 'amlodipine plus olmesartan’:ti,ab OR 'alea':ti,ab OR 'amelior':ti,ab OR 'amlodipine besylate olmesartan medoxomil':ti,ab OR 'amlodipine plus olmesartan':ti,ab OR 'amlodipine/olmesartan':ti,ab OR 'azor':ti,ab OR 'balzak':ti,ab OR 'bivis':ti,ab OR 'capenon':ti,ab OR 'forzaten':ti,ab OR 'olmesartan medoxomil plus amlodipine':ti,ab OR 'olmesartan medoxomil/amlodipine besylate':ti,ab OR 'olmesartan plus amlodipine':ti,ab OR 'olmesartan/amlodipine':ti,ab OR 'sevikar':ti,ab OR 'vocado':ti,ab OR 'amlodipine plus valsartan'/de OR 'amlodipine plus valsartan’:ti,ab OR 'amlodipine besylate/valsartan':ti,ab OR 'amlodipine plus valsartan':ti,ab OR 'amlodipine valsartan':ti,ab OR 'copalia':ti,ab OR 'dafiro':ti,ab OR 'exforge':ti,ab OR 'imprida':ti,ab OR 'valsartan plus amlodipine':ti,ab OR 'valsartan/amlodipine':ti,ab OR 'amoxicillin plus clavulanic acid'/de OR 'amoxicillin plus clavulanic acid’:ti,ab OR 'aclam':ti,ab OR 'aktil':ti,ab OR 'amocla':ti,ab OR 'amoclan':ti,ab OR 'amoclav':ti,ab OR 'amoksiklav':ti,ab OR 'amox clav':ti,ab OR 'amoxi clavulanate':ti,ab OR 'amoxicillin and clavulanate potassium':ti,ab OR 'amoxicillin clavulanic acid':ti,ab OR 'amoxicillin plus clavulanate potassium':ti,ab OR 'amoxicillin plus clavulanic acid':ti,ab OR 'amoxicillin potassium clavulanate':ti,ab OR 'amoxicillin/clavulanate potassium':ti,ab OR 'amoxiclav':ti,ab OR 'amoxycillin clavulanic acid':ti,ab OR 'ancla':ti,ab OR 'augmentan':ti,ab OR 'augmentin':ti,ab OR 'augmentine':ti,ab OR 'bioclavid':ti,ab OR 'brl 25000':ti,ab OR 'brl25000':ti,ab OR 'cavumox':ti,ab OR 'ciblor':ti,ab OR 'clamax':ti,ab OR 'clamoxin':ti,ab OR 'clavam':ti,ab OR 'clavamox':ti,ab OR 'clavubactin':ti,ab OR 'clavucid':ti,ab OR 'clavulanate potassium/amoxicillin':ti,ab OR 'clavulanate potentiated amoxycillin':ti,ab OR 'clavulanic acid/amoxicillin':ti,ab OR 'clavulin':ti,ab OR 'co amoxiclav':ti,ab OR 'co amoxyclav':ti,ab OR 'coamoxiclav':ti,ab OR 'coamoxyclav':ti,ab OR 'curam':ti,ab OR 'duomox':ti,ab OR 'enhancin':ti,ab OR 'fleming':ti,ab OR 'forcid':ti,ab OR 'forcid solutab':ti,ab OR 'moxiclav':ti,ab OR 'spektramox':ti,ab OR 'stacillin':ti,ab OR 'strenzen':ti,ab OR 'synulox':ti,ab OR 'taromentin':ti,ab OR 'xiclav':ti,ab OR 'cilastatin plus imipenem'/de OR 'cilastatin plus imipenem’:ti,ab OR 'cilastatin plus imipenem':ti,ab OR 'cilastatin sodium/imipenem':ti,ab OR 'cilastatin, imipenem':ti,ab OR 'imipenem cilastatin':ti,ab OR 'imipenem plus cilastatin':ti,ab OR 'mk 0787 mk 0791 mixture':ti,ab OR 'mk 787 mk 791 mixture':ti,ab OR 'prepenem':ti,ab OR 'primaxin':ti,ab OR 'tenacid':ti,ab OR 'thienam':ti,ab OR 'tienam':ti,ab OR 'tienem':ti,ab OR 'zienam':ti,ab OR 'efavirenz plus emtricitabine plus tenofovir disoproxil'/de OR 'efavirenz plus emtricitabine plus tenofovir disoproxil’:ti,ab OR 'atripla':ti,ab OR 'efavirenz/emtricitabine/tenofovir disoproxil':ti,ab OR 'efavirenz/tenofovir disoproxil fumarate/emtricitabine':ti,ab OR 'emtricitabine/tenofovir disoproxil fumarate/efavirenz':ti,ab OR 'emtricitabine/tenofovir disoproxil/efavirenz':ti,ab OR 'tenofovir disoproxil fumarate/emtricitabine/efavirenz':ti,ab OR 'tenofovir disoproxil/emtricitabine/efavirenz':ti,ab OR 'cobicistat plus elvitegravir plus emtricitabine plus tenofovir disoproxil'/de OR 'cobicistat plus elvitegravir plus emtricitabine plus tenofovir disoproxil’:ti,ab OR 'elvitegravir cobicistat emtricitabine tenofovir disoproxil fumarate':ti,ab OR 'elvitegravir, cobicistat, emtricitabine, and tenofovir disoproxil fumarate':ti,ab OR 'elvitegravir/cobicistat/emtricitabine/tenofovir df':ti,ab OR 'elvitegravir/cobicistat/emtricitabine/tenofovir disoproxil fumarate':ti,ab OR 'genvoya':ti,ab OR 'quad pill':ti,ab OR 'stribild':ti,ab OR 'emtricitabine plus rilpivirine plus tenofovir disoproxil'/de OR 'emtricitabine plus rilpivirine plus tenofovir disoproxil’:ti,ab OR 'complera':ti,ab OR 'emtricitabine rilpivirine tenofovir':ti,ab OR 'emtricitabine/rilpivirine/tenofovir disoproxil fumarate':ti,ab OR 'emtricitabine/tenofovir disoproxil fumarate/rilpivirine':ti,ab OR 'emtricitabine/tenofovir disoproxil/rilpivirine':ti,ab OR 'eviplera':ti,ab OR 'rilpivirine/emtricitabine/tenofovir disoproxil':ti,ab OR 'rilpivirine/tenofovir disoproxil fumarate/emtricitabine':ti,ab OR 'tenofovir disoproxil fumarate/emtricitabine/rilpivirine':ti,ab OR 'tenofovir disoproxil/emtricitabine/rilpivirine':ti,ab OR 'emtricitabine plus tenofovir disoproxil'/de OR 'emtricitabine plus tenofovir disoproxil’:ti,ab OR 'emtricitabine plus tenofovir disoproxil':ti,ab OR 'emtricitabine tenofovir disoproxil fumarate':ti,ab OR 'emtricitabine/tenofovir disoproxil':ti,ab OR 'emtriva-viread':ti,ab OR 'tenofovir disoproxil fumarate plus emtricitabine':ti,ab OR 'tenofovir disoproxil fumarate/emtricitabine':ti,ab OR 'tenofovir disoproxil plus emtricitabine':ti,ab OR 'tenofovir disoproxil/emtricitabine':ti,ab OR 'torad':ti,ab OR 'truvada':ti,ab OR 'piperacillin plus tazobactam'/de OR 'piperacillin plus tazobactam’:ti,ab OR 'piperacillin'/de OR 'piperacillin and tazobactam':ti,ab OR 'piperacillin plus tazobactam':ti,ab OR 'piperacillin sodium plus tazobactam':ti,ab OR 'piperacillin tazobactam':ti,ab OR 'pipercillin sodium tazobactam sodium':ti,ab OR 'tazip':ti,ab OR 'tazobactam plus piperacillin':ti,ab OR 'tazobactam, piperacillin':ti,ab OR 'tazocel':ti,ab OR 'tazocillin':ti,ab OR 'tazocilline':ti,ab OR 'tazocin':ti,ab OR 'tazonam':ti,ab OR 'tazorex':ti,ab OR 'yp 14':ti,ab OR 'yp 18':ti,ab OR 'yp14':ti,ab OR 'yp18':ti,ab OR 'zobactam':ti,ab OR 'zosyn':ti,ab OR 'metformin plus sitagliptin'/de OR 'metformin plus sitagliptin’:ti,ab OR 'efficib':ti,ab OR 'janumet':ti,ab OR 'metformin plus sitagliptin':ti,ab OR 'metformin-sitagliptin':ti,ab OR 'mk 0431a':ti,ab OR 'mk0431a':ti,ab OR 'ristfor':ti,ab OR 'sitagliptin phosphate metformin hydrochloride':ti,ab OR 'sitagliptin plus metformin':ti,ab OR 'sitagliptin-metformin':ti,ab OR 'velmetia':ti,ab OR 'dexamethasone plus tobramycin'/de OR 'dexamethasone plus tobramycin’:ti,ab OR 'dexamethasone plus tobramycin':ti,ab OR 'dexamethasone-tobramycin':ti,ab OR 'tobradex':ti,ab OR 'tobramycin'/de OR 'tobramycin and dexamethasone':ti,ab OR 'tobramycin-dexamethasone':ti,ab OR 'cotrimoxazole'/de OR 'cotrimoxazole’:ti,ab OR 'abactrim':ti,ab OR 'alfatrim':ti,ab OR 'apo sulfatrim':ti,ab OR 'bactar':ti,ab OR 'bactifor':ti,ab OR 'bactramin':ti,ab OR 'bactrim':ti,ab OR 'bactrimel':ti,ab OR 'bethaprim':ti,ab OR 'biseptol':ti,ab OR 'biseptol480':ti,ab OR 'centran':ti,ab OR 'centrin':ti,ab OR 'chemotrim':ti,ab OR 'co trimoxazole':ti,ab OR 'comox':ti,ab OR 'cotrim':ti,ab OR 'cotrimoxazole':ti,ab OR 'co-trimoxazole':ti,ab OR 'deprim':ti,ab OR 'drylin':ti,ab OR 'duratrimet':ti,ab OR 'eltrianyl':ti,ab OR 'escoprim':ti,ab OR 'eslectin':ti,ab OR 'espectrin':ti,ab OR 'eusaprim':ti,ab OR 'fectrim':ti,ab OR 'groprim':ti,ab OR 'helveprim':ti,ab OR 'imexim':ti,ab OR 'kepinol':ti,ab OR 'lagatrim':ti,ab OR 'lescot':ti,ab OR 'linaris':ti,ab OR 'microtrim':ti,ab OR 'nopil':ti,ab OR 'oecotrim':ti,ab OR 'omsat':ti,ab OR 'oriprim':ti,ab OR 'potesept':ti,ab OR 'resprim':ti,ab OR 'ro 6 2580-11':ti,ab OR 'ro 62580':ti,ab OR 'septra':ti,ab OR 'septran':ti,ab OR 'septrim':ti,ab OR 'septrin':ti,ab OR 'septrine':ti,ab OR 'sigaprim':ti,ab OR 'soltrim':ti,ab OR 'sulfamethoprim':ti,ab OR 'sulfamethoprim-ds':ti,ab OR 'sulfamethoxazole'/de OR 'sulfamethoxazole and trimethoprim':ti,ab OR 'sulfamethoxazole plus trimethoprim':ti,ab OR 'sulfamethoxazole trimethoprim':ti,ab OR 'sulfaprim':ti,ab OR 'sulfatrim':ti,ab OR 'sulfatrim-ds':ti,ab OR 'sulfatrim-ss':ti,ab OR 'sulfotrim':ti,ab OR 'sulmeprim':ti,ab OR 'sulprim':ti,ab OR 'sumetrolim':ti,ab OR 'sumetrolin':ti,ab OR 'supracombin':ti,ab OR 'thiocuran':ti,ab OR 'tmp smx':ti,ab OR 'tms forte':ti,ab OR 'trib':ti,ab OR 'trigonyl':ti,ab OR 'trimedin':ti,ab OR 'trimeth/sulfa':ti,ab OR 'trimethoprim plus sulfamethoxazole':ti,ab OR 'trimethoprim sulfamethoxazole':ti,ab OR 'trimethoprimsulfa':ti,ab OR 'trimethoprimsulfamethoxazole':ti,ab OR 'trimethoprim-sulfamethoxazole':ti,ab OR 'trimetoprim-sulfa':ti,ab OR 'trimetoprimsulfamethoxazole':ti,ab OR 'trimezol':ti,ab OR 'trimforte':ti,ab OR 'trimosulfa':ti,ab OR 'trimoxazole':ti,ab OR 'uro ts d':ti,ab OR 'uroplus ds':ti,ab OR 'uroplus ss':ti,ab

**Scopus – limited to English language; 118 results; 144 results on 9.2.2024**

TITLE-ABS({acute kidney injury} OR {acute kidney injuries} OR {acute renal injury} OR {acute renal injuries} OR {acute renal insufficiency} OR {acute renal insufficiencies} OR {acute kidney insufficiency} OR {acute kidney insufficiencies} OR {acute kidney failure} OR {acute kidney failures} OR {acute renal failure} OR {acute renal failures} OR {acute kidney damage} OR {acute kidney damages} OR {acute renal damage} OR {acute renal damages})

AND

TITLE-ABS({nephrotoxicity} OR {nephrotoxicities} OR {nephrotoxic} OR {nephrotoxin} OR {nephrotoxins} OR {nephro toxic} OR {nephro toxin} OR {nephro toxins} OR {nephro toxicity} OR {nephro toxicities} OR {kidney toxic} OR {kidney toxicity} OR {kidney toxicities} OR {kidney toxin} OR {kidney toxins} OR {renal toxic} OR {renal toxicity} OR {renal toxicities} OR {renal toxin} OR {renal toxins})

AND

TITLE-ABS({combination drug therapy} OR {combination drug therapies} OR {drug polytherapy} OR {drug polytherapies} OR {drug combination} OR {drug combinations} OR {combination therapy} OR {combination therapies} OR {drug combination therapy} OR {drug combination therapies} OR {medication combination} OR {medication combinations} OR {combination medication} OR {combination medications} OR {combined medication} OR {combined medications} OR {combined medicine} OR {combined medicines} OR {combination drug regimen} OR {combination drug regimens} OR {combined drug regimen} OR { combined drug regimens} OR {multi-drug regimen} OR {multi-drug regimens} OR {multidrug regimen} OR {multidrug regimens} OR {multiple drug regimen} OR {multiple drug regimens} OR {combination drug treatment} OR {combination drug treatments} OR {combination pharmacotherapy} OR {combination pharmacotherapies} OR {combined pharmacotherapy} OR {combined pharmacotherapies} OR {combined drug therapy} OR {combined drug therapies} OR {combined drug treatment} OR {combined drug treatments} OR {concomitant drug therapy} OR {concomitant drug therapies} OR {concomitant drug} OR {concomitant drugs} OR {concomitant medication} OR {concomitant medications} OR {drug burden} OR {drug burdens} OR {drug exposure} OR {drug exposures} OR {medication exposure} OR {medication exposures} OR {drug intensity} OR {drug intensities} OR {prescription drug} OR {prescription drugs} OR {alea} OR {amelior} OR {Amlodipine Besylate Olmesartan Medoxomil} OR {amlodipine plus Olmesartan} OR {amlodipine/Olmesartan} OR {Azor} OR {balzak} OR {bivis} OR {capenon} OR {forzaten} OR {olmesartan medoxomil plus amlodipine} OR {olmesartan medoxomil/amlodipine besylate} OR {olmesartan plus amlodipine} OR {olmesartan/amlodipine} OR {sevikar} OR {vocado} OR {amlodipine besylate/valsartan} OR {amlodipine plus valsartan} OR {Amlodipine Valsartan} OR {copalia} OR {dafiro} OR {Exforge} OR {imprida} OR {valsartan plus amlodipine} OR {valsartan/amlodipine} OR {aclam} OR {aktil} OR {amocla} OR {amoclan} OR {amoclav} OR {amoksiklav} OR {Amox clav} OR {Amoxi Clavulanate} OR {amoxicillin and clavulanate potassium} OR {Amoxicillin Clavulanic Acid} OR {amoxicillin plus clavulanate potassium} OR {amoxicillin plus clavulanic acid} OR {Amoxicillin Potassium Clavulanate} OR {amoxicillin/clavulanate potassium} OR {amoxiclav} OR {Amoxycillin Clavulanic Acid} OR {ancla} OR {augmentan} OR {Augmentin} OR {augmentine} OR {bioclavid} OR {BRL 25000} OR {BRL25000} OR {cavumox} OR {ciblor} OR {clamax} OR {clamoxin} OR {clavam} OR {clavamox} OR {clavubactin} OR {clavucid} OR {clavulanate potassium/amoxicillin} OR {Clavulanate Potentiated Amoxycillin} OR {clavulanic acid/amoxicillin} OR {Clavulin} OR {Co amoxiclav} OR {co amoxyclav} OR {Coamoxiclav} OR {coamoxyclav} OR {curam} OR {duomox} OR {enhancin} OR {fleming} OR {forcid} OR {forcid solutab} OR {moxiclav} OR {spektramox} OR {stacillin} OR {strenzen} OR {Synulox} OR {taromentin} OR {xiclav} OR {cilastatin plus imipenem} OR {cilastatin sodium/imipenem} OR {Cilastatin, Imipenem} OR {Imipenem Cilastatin} OR {imipenem plus cilastatin} OR {MK 0787 MK 0791 mixture} OR {MK 787 MK 791 mixture} OR {prepenem} OR {Primaxin} OR {tenacid} OR {Thienam} OR {tienam} OR {tienem} OR {Zienam} OR {Atripla} OR {efavirenz/emtricitabine/tenofovir disoproxil} OR {efavirenz/tenofovir disoproxil fumarate/emtricitabine} OR {emtricitabine/tenofovir disoproxil fumarate/efavirenz} OR {emtricitabine/tenofovir disoproxil/efavirenz} OR {tenofovir disoproxil fumarate/emtricitabine/efavirenz} OR {tenofovir disoproxil/emtricitabine/efavirenz} OR {Elvitegravir Cobicistat Emtricitabine Tenofovir Disoproxil Fumarate} OR {Elvitegravir, Cobicistat, Emtricitabine, and Tenofovir Disoproxil Fumarate} OR {elvitegravir/cobicistat/emtricitabine/tenofovir df} OR {elvitegravir/cobicistat/emtricitabine/tenofovir disoproxil fumarate} OR {Genvoya} OR {Quad Pill} OR {stribild} OR {Complera} OR {Emtricitabine Rilpivirine Tenofovir} OR {emtricitabine/rilpivirine/tenofovir disoproxil fumarate} OR {emtricitabine/tenofovir disoproxil fumarate/rilpivirine} OR {emtricitabine/tenofovir disoproxil/rilpivirine} OR {eviplera} OR {rilpivirine/emtricitabine/tenofovir disoproxil} OR {rilpivirine/tenofovir disoproxil fumarate/emtricitabine} OR {tenofovir disoproxil fumarate/emtricitabine/rilpivirine} OR {tenofovir disoproxil/emtricitabine/rilpivirine} OR {emtricitabine plus tenofovir disoproxil} OR {Emtricitabine Tenofovir Disoproxil Fumarate} OR {emtricitabine/tenofovir disoproxil} OR {emtriva-viread} OR {tenofovir disoproxil fumarate plus emtricitabine} OR {tenofovir disoproxil fumarate/emtricitabine} OR {tenofovir disoproxil plus emtricitabine} OR {tenofovir disoproxil/emtricitabine} OR {Torad} OR {Truvada} OR {piperacillin and tazobactam} OR {piperacillin plus tazobactam} OR {piperacillin sodium plus tazobactam} OR {Piperacillin Tazobactam} OR {Pipercillin Sodium Tazobactam Sodium} OR {tazip} OR {tazobactam plus piperacillin} OR {Tazobactam, Piperacillin} OR {Tazocel} OR {Tazocillin} OR {tazocilline} OR {Tazocin} OR {tazonam} OR {tazorex} OR {yp 14} OR {yp 18} OR {yp14} OR {yp18} OR {zobactam} OR {Zosyn} OR {efficib} OR {Janumet} OR {metformin plus sitagliptin} OR {metformin-sitagliptin} OR {mk 0431a} OR {mk0431a} OR {ristfor} OR {Sitagliptin Phosphate Metformin Hydrochloride} OR {sitagliptin plus metformin} OR {sitagliptin-metformin} OR {velmetia} OR {dexamethasone plus tobramycin} OR {dexamethasone-tobramycin} OR {TobraDex} OR {tobramycin and dexamethasone} OR {tobramycin-dexamethasone} OR {Abactrim} OR {alfatrim} OR {apo sulfatrim} OR {bactar} OR {Bactifor} OR {bactramin} OR {Bactrim} OR {bactrimel} OR {bethaprim} OR {Biseptol} OR {Biseptol480} OR {Centran} OR {Centrin} OR {chemotrim} OR {Co Trimoxazole} OR {comox} OR {cotrim} OR {Cotrimoxazole} OR {co-trimoxazole} OR {deprim} OR {Drylin} OR {duratrimet} OR {eltrianyl} OR {escoprim} OR {Eslectin} OR {espectrin} OR {Eusaprim} OR {fectrim} OR {groprim} OR {helveprim} OR {imexim} OR {Kepinol} OR {lagatrim} OR {Lescot} OR {linaris} OR {microtrim} OR {nopil} OR {oecotrim} OR {omsat} OR {Oriprim} OR {potesept} OR {resprim} OR {ro 6 2580-11} OR {ro 62580} OR {Septra} OR {septran} OR {septrim} OR {Septrin} OR {septrine} OR {sigaprim} OR {soltrim} OR {sulfamethoprim} OR {sulfamethoprim-ds} OR {sulfamethoxazole and trimethoprim} OR {sulfamethoxazole plus trimethoprim} OR {sulfamethoxazole trimethoprim} OR {sulfaprim} OR {sulfatrim} OR {sulfatrim-ds} OR {sulfatrim-ss} OR {sulfotrim} OR {sulmeprim} OR {Sulprim} OR {sumetrolim} OR {sumetrolin} OR {supracombin} OR {thiocuran} OR {TMP SMX} OR {tms forte} OR {trib} OR {trigonyl} OR {Trimedin} OR {trimeth/sulfa} OR {trimethoprim plus sulfamethoxazole} OR {Trimethoprim Sulfamethoxazole} OR {Trimethoprimsulfa} OR {trimethoprimsulfamethoxazole} OR {trimethoprim-sulfamethoxazole} OR {trimetoprim-sulfa} OR {trimetoprimsulfamethoxazole} OR {trimezol} OR {trimforte} OR {Trimosulfa} OR {trimoxazole} OR {uro ts d} OR {uroplus ds} OR {uroplus ss})

**Web of Science – limited to English language; 160 results; 199 results on 9.2.2024**

TI=("acute kidney injur*") OR AB=("acute kidney injur*") OR TI=("acute renal injur*") OR AB=("acute renal injur*") OR TI=("acute renal insufficienc*") OR AB=("acute renal insufficienc*") OR TI=("acute kidney insufficienc*") OR AB=("acute kidney insufficienc*") OR TI=("acute kidney failure*") OR AB=("acute kidney failure*") OR TI=("acute renal failure*") OR AB=("acute renal failure*") OR TI=("acute kidney damage*") OR AB=("acute kidney damage*") OR TI=("acute renal damage*") OR AB=("acute renal damage*")

AND

TI=("nephrotoxi*") OR AB=("nephrotoxi*") OR TI=("nephro toxi*") OR AB=("nephro toxi*") OR TI=("kidney toxi*") OR AB=("kidney toxi*") OR TI=("renal toxi*") OR AB=("renal toxi*")

AND

TI=("combination drug therap*") OR AB=("combination drug therap*") OR TI=("drug polytherap*") OR AB=("drug polytherap*") OR TI=("drug combination*") OR AB=("drug combination*") OR TI=("combination therap*") OR AB=("combination therap*") OR TI=("drug combination therap*") OR AB=("drug combination therap*") OR TI=("medication combination*") OR AB=("medication combination*") OR TI=("combination medic*") OR AB=("combination medic*") OR TI=("combined medic*") OR AB=("combined medic*") OR TI=("combination drug regimen*") OR AB=("combination drug regimen*") OR TI=("combined drug regimen*") OR AB=("combined drug regimen*") OR TI=("multi-drug regimen*") OR AB=("multi-drug regimen*") OR TI=("multidrug regimen*") OR AB=("multidrug regimen*") OR TI=("multiple drug regimen*") OR AB=("multiple drug regimen*") OR TI=("combination drug treatment*") OR AB=("combination drug treatment*") OR TI=("combination pharmacotherap*") OR AB=("combination pharmacotherap*") OR TI=("combined pharmacotherap*") OR AB=("combined pharmacotherap*") OR TI=("combined drug therap*") OR AB=("combined drug therap*") OR TI=("combined drug treatment*") OR AB=("combined drug treatment*") OR TI=("concomitant drug therap*") OR AB=("concomitant drug therap*") OR TI=("concomitant drug*") OR AB=("concomitant drug*") OR TI=("concomitant medication*") OR AB=("concomitant medication*") OR TI=("drug burden*") OR AB=("drug burden*") OR TI=("drug exposure*") OR AB=("drug exposure*") OR TI=("medication exposure*") OR AB=("medication exposure*") OR TI=("drug intensit*") OR AB=("drug intensit*") OR TI=(“prescription drug*”) OR AB=(“prescription drug*”) OR TI=(alea) OR AB=(alea) OR TI=(amelior) OR AB=(amelior) OR TI=("Amlodipine Besylate Olmesartan Medoxomil") OR AB=("Amlodipine Besylate Olmesartan Medoxomil") OR TI=("amlodipine plus Olmesartan") OR AB=("amlodipine plus Olmesartan") OR TI=("amlodipine/Olmesartan") OR AB=("amlodipine/Olmesartan") OR TI=(Azor) OR AB=(Azor) OR TI=(balzak) OR AB=(balzak) OR TI=(bivis) OR AB=(bivis) OR TI=(capenon) OR AB=(capenon) OR TI=(forzaten) OR AB=(forzaten) OR TI=("olmesartan medoxomil plus amlodipine") OR AB=("olmesartan medoxomil plus amlodipine") OR TI=("olmesartan medoxomil/amlodipine besylate") OR AB=("olmesartan medoxomil/amlodipine besylate") OR TI=("olmesartan plus amlodipine") OR AB=("olmesartan plus amlodipine") OR TI=("olmesartan/amlodipine") OR AB=("olmesartan/amlodipine") OR TI=(sevikar) OR AB=(sevikar) OR TI=(vocado) OR AB=(vocado) OR TI=("amlodipine besylate/valsartan") OR AB=("amlodipine besylate/valsartan") OR TI=("amlodipine plus valsartan") OR AB=("amlodipine plus valsartan") OR TI=("Amlodipine Valsartan") OR AB=("Amlodipine Valsartan") OR TI=(copalia) OR AB=(copalia) OR TI=(dafiro) OR AB=(dafiro) OR TI=(Exforge) OR AB=(Exforge) OR TI=(imprida) OR AB=(imprida) OR TI=("valsartan plus amlodipine") OR AB=("valsartan plus amlodipine") OR TI=("valsartan/amlodipine") OR AB=("valsartan/amlodipine") OR TI=(aclam) OR AB=(aclam) OR TI=(aktil) OR AB=(aktil) OR TI=(amocla) OR AB=(amocla) OR TI=(amoclan) OR AB=(amoclan) OR TI=(amoclav) OR AB=(amoclav) OR TI=(amoksiklav) OR AB=(amoksiklav) OR TI=("Amox clav") OR AB=("Amox clav") OR TI=("Amoxi Clavulanate") OR AB=("Amoxi Clavulanate") OR TI=("amoxicillin and clavulanate potassium") OR AB=("amoxicillin and clavulanate potassium") OR TI=("Amoxicillin Clavulanic Acid") OR AB=("Amoxicillin Clavulanic Acid") OR TI=("amoxicillin plus clavulanate potassium") OR AB=("amoxicillin plus clavulanate potassium") OR TI=("amoxicillin plus clavulanic acid") OR AB=("amoxicillin plus clavulanic acid") OR TI=("Amoxicillin Potassium Clavulanate") OR AB=("Amoxicillin Potassium Clavulanate") OR TI=("amoxicillin/clavulanate potassium") OR AB=("amoxicillin/clavulanate potassium") OR TI=(amoxiclav) OR AB=(amoxiclav) OR TI=("Amoxycillin Clavulanic Acid") OR AB=("Amoxycillin Clavulanic Acid") OR TI=(ancla) OR AB=(ancla) OR TI=(augmentan) OR AB=(augmentan) OR TI=(Augmentin) OR AB=(Augmentin) OR TI=(augmentine) OR AB=(augmentine) OR TI=(bioclavid) OR AB=(bioclavid) OR TI=("BRL 25000") OR AB=("BRL 25000") OR TI=(BRL25000) OR AB=(BRL25000) OR TI=(cavumox) OR AB=(cavumox) OR TI=(ciblor) OR AB=(ciblor) OR TI=(clamax) OR AB=(clamax) OR TI=(clamoxin) OR AB=(clamoxin) OR TI=(clavam) OR AB=(clavam) OR TI=(clavamox) OR AB=(clavamox) OR TI=(clavubactin) OR AB=(clavubactin) OR TI=(clavucid) OR AB=(clavucid) OR TI=("clavulanate potassium/amoxicillin") OR AB=("clavulanate potassium/amoxicillin") OR TI=("Clavulanate Potentiated Amoxycillin") OR AB=("Clavulanate Potentiated Amoxycillin") OR TI=("clavulanic acid/amoxicillin") OR AB=("clavulanic acid/amoxicillin") OR TI=(Clavulin) OR AB=(Clavulin) OR TI=("Co amoxiclav") OR AB=("Co amoxiclav") OR TI=("co amoxyclav") OR AB=("co amoxyclav") OR TI=(Coamoxiclav) OR AB=(Coamoxiclav) OR TI=(coamoxyclav) OR AB=(coamoxyclav) OR TI=(curam) OR AB=(curam) OR TI=(duomox) OR AB=(duomox) OR TI=(enhancin) OR AB=(enhancin) OR TI=(fleming) OR AB=(fleming) OR TI=(forcid) OR AB=(forcid) OR TI=("forcid solutab") OR AB=("forcid solutab") OR TI=(moxiclav) OR AB=(moxiclav) OR TI=(spektramox) OR AB=(spektramox) OR TI=(stacillin) OR AB=(stacillin) OR TI=(strenzen) OR AB=(strenzen) OR TI=(Synulox) OR AB=(Synulox) OR TI=(taromentin) OR AB=(taromentin) OR TI=(xiclav) OR AB=(xiclav) OR TI=("cilastatin plus imipenem") OR AB=("cilastatin plus imipenem") OR TI=("cilastatin sodium/imipenem") OR AB=("cilastatin sodium/imipenem") OR TI=("Cilastatin, Imipenem") OR AB=("Cilastatin, Imipenem") OR TI=("Imipenem Cilastatin") OR AB=("Imipenem Cilastatin") OR TI=("imipenem plus cilastatin") OR AB=("imipenem plus cilastatin") OR TI=("MK 0787 MK 0791 mixture") OR AB=("MK 0787 MK 0791 mixture") OR TI=("MK 787 MK 791 mixture") OR AB=("MK 787 MK 791 mixture") OR TI=(prepenem) OR AB=(prepenem) OR TI=(Primaxin) OR AB=(Primaxin) OR TI=(tenacid) OR AB=(tenacid) OR TI=(Thienam) OR AB=(Thienam) OR TI=(tienam) OR AB=(tienam) OR TI=(tienem) OR AB=(tienem) OR TI=(Zienam) OR AB=(Zienam) OR TI=(Atripla) OR AB=(Atripla) OR TI=("efavirenz/emtricitabine/tenofovir disoproxil") OR AB=("efavirenz/emtricitabine/tenofovir disoproxil") OR TI=("efavirenz/tenofovir disoproxil fumarate/emtricitabine") OR AB=("efavirenz/tenofovir disoproxil fumarate/emtricitabine") OR TI=("emtricitabine/tenofovir disoproxil fumarate/efavirenz") OR AB=("emtricitabine/tenofovir disoproxil fumarate/efavirenz") OR TI=("emtricitabine/tenofovir disoproxil/efavirenz") OR AB=("emtricitabine/tenofovir disoproxil/efavirenz") OR TI=("tenofovir disoproxil fumarate/emtricitabine/efavirenz") OR AB=("tenofovir disoproxil fumarate/emtricitabine/efavirenz") OR TI=("tenofovir disoproxil/emtricitabine/efavirenz") OR AB=("tenofovir disoproxil/emtricitabine/efavirenz") OR TI=("Elvitegravir Cobicistat Emtricitabine Tenofovir Disoproxil Fumarate") OR AB=("Elvitegravir Cobicistat Emtricitabine Tenofovir Disoproxil Fumarate") OR TI=("Elvitegravir, Cobicistat, Emtricitabine, and Tenofovir Disoproxil Fumarate") OR AB=("Elvitegravir, Cobicistat, Emtricitabine, and Tenofovir Disoproxil Fumarate") OR TI=("elvitegravir/cobicistat/emtricitabine/tenofovir df") OR AB=("elvitegravir/cobicistat/emtricitabine/tenofovir df") OR TI=("elvitegravir/cobicistat/emtricitabine/tenofovir disoproxil fumarate") OR AB=("elvitegravir/cobicistat/emtricitabine/tenofovir disoproxil fumarate") OR TI=(Genvoya) OR AB=(Genvoya) OR TI=("Quad Pill") OR AB=("Quad Pill") OR TI=(stribild) OR AB=(stribild) OR TI=(Complera) OR AB=(Complera) OR TI=("Emtricitabine Rilpivirine Tenofovir") OR AB=("Emtricitabine Rilpivirine Tenofovir") OR TI=("emtricitabine/rilpivirine/tenofovir disoproxil fumarate") OR AB=("emtricitabine/rilpivirine/tenofovir disoproxil fumarate") OR TI=("emtricitabine/tenofovir disoproxil fumarate/rilpivirine") OR AB=("emtricitabine/tenofovir disoproxil fumarate/rilpivirine") OR TI=("emtricitabine/tenofovir disoproxil/rilpivirine") OR AB=("emtricitabine/tenofovir disoproxil/rilpivirine") OR TI=(eviplera) OR AB=(eviplera) OR TI=("rilpivirine/emtricitabine/tenofovir disoproxil") OR AB=("rilpivirine/emtricitabine/tenofovir disoproxil") OR TI=("rilpivirine/tenofovir disoproxil fumarate/emtricitabine") OR AB=("rilpivirine/tenofovir disoproxil fumarate/emtricitabine") OR TI=("tenofovir disoproxil fumarate/emtricitabine/rilpivirine") OR AB=("tenofovir disoproxil fumarate/emtricitabine/rilpivirine") OR TI=("tenofovir disoproxil/emtricitabine/rilpivirine") OR AB=("tenofovir disoproxil/emtricitabine/rilpivirine") OR TI=("emtricitabine plus tenofovir disoproxil") OR AB=("emtricitabine plus tenofovir disoproxil") OR TI=("Emtricitabine Tenofovir Disoproxil Fumarate") OR AB=("Emtricitabine Tenofovir Disoproxil Fumarate") OR TI=("emtricitabine/tenofovir disoproxil") OR AB=("emtricitabine/tenofovir disoproxil") OR TI=("emtriva-viread") OR AB=("emtriva-viread") OR TI=("tenofovir disoproxil fumarate plus emtricitabine") OR AB=("tenofovir disoproxil fumarate plus emtricitabine") OR TI=("tenofovir disoproxil fumarate/emtricitabine") OR AB=("tenofovir disoproxil fumarate/emtricitabine") OR TI=("tenofovir disoproxil plus emtricitabine") OR AB=("tenofovir disoproxil plus emtricitabine") OR TI=("tenofovir disoproxil/emtricitabine") OR AB=("tenofovir disoproxil/emtricitabine") OR TI=(Torad) OR AB=(Torad) OR TI=(Truvada) OR AB=(Truvada) OR TI=("piperacillin and tazobactam") OR AB=("piperacillin and tazobactam") OR TI=("piperacillin plus tazobactam") OR AB=("piperacillin plus tazobactam") OR TI=("piperacillin sodium plus tazobactam") OR AB=("piperacillin sodium plus tazobactam") OR TI=("Piperacillin Tazobactam") OR AB=("Piperacillin Tazobactam") OR TI=("Pipercillin Sodium Tazobactam Sodium") OR AB=("Pipercillin Sodium Tazobactam Sodium") OR TI=(tazip) OR AB=(tazip) OR TI=("tazobactam plus piperacillin") OR AB=("tazobactam plus piperacillin") OR TI=("Tazobactam, Piperacillin") OR AB=("Tazobactam, Piperacillin") OR TI=(Tazocel) OR AB=(Tazocel) OR TI=(Tazocillin) OR AB=(Tazocillin) OR TI=(tazocilline) OR AB=(tazocilline) OR TI=(Tazocin) OR AB=(Tazocin) OR TI=(tazonam) OR AB=(tazonam) OR TI=(tazorex) OR AB=(tazorex) OR TI=("yp 14") OR AB=("yp 14") OR TI=("yp 18") OR AB=("yp 18") OR TI=(yp14) OR AB=(yp14) OR TI=(yp18) OR AB=(yp18) OR TI=(zobactam) OR AB=(zobactam) OR TI=(Zosyn) OR AB=(Zosyn) OR TI=(efficib) OR AB=(efficib) OR TI=(Janumet) OR AB=(Janumet) OR TI=("metformin plus sitagliptin") OR AB=("metformin plus sitagliptin") OR TI=("metformin-sitagliptin") OR AB=("metformin-sitagliptin") OR TI=("mk 0431a") OR AB=("mk 0431a") OR TI=(mk0431a) OR AB=(mk0431a) OR TI=(ristfor) OR AB=(ristfor) OR TI=("Sitagliptin Phosphate Metformin Hydrochloride") OR AB=("Sitagliptin Phosphate Metformin Hydrochloride") OR TI=("sitagliptin plus metformin") OR AB=("sitagliptin plus metformin") OR TI=("sitagliptin-metformin") OR AB=("sitagliptin-metformin") OR TI=(velmetia) OR AB=(velmetia) OR TI=("dexamethasone plus tobramycin") OR AB=("dexamethasone plus tobramycin") OR TI=("dexamethasone-tobramycin") OR AB=("dexamethasone-tobramycin") OR TI=(TobraDex) OR AB=(TobraDex) OR TI=("tobramycin and dexamethasone") OR AB=("tobramycin and dexamethasone") OR TI=("tobramycin-dexamethasone") OR AB=("tobramycin-dexamethasone") OR TI=(Abactrim) OR AB=(Abactrim) OR TI=(alfatrim) OR AB=(alfatrim) OR TI=("apo sulfatrim") OR AB=("apo sulfatrim") OR TI=(bactar) OR AB=(bactar) OR TI=(Bactifor) OR AB=(Bactifor) OR TI=(bactramin) OR AB=(bactramin) OR TI=(Bactrim) OR AB=(Bactrim) OR TI=(bactrimel) OR AB=(bactrimel) OR TI=(bethaprim) OR AB=(bethaprim) OR TI=(Biseptol) OR AB=(Biseptol) OR TI=(Biseptol480) OR AB=(Biseptol480) OR TI=(Centran) OR AB=(Centran) OR TI=(Centrin) OR AB=(Centrin) OR TI=(chemotrim) OR AB=(chemotrim) OR TI=("Co Trimoxazole") OR AB=("Co Trimoxazole") OR TI=(comox) OR AB=(comox) OR TI=(cotrim) OR AB=(cotrim) OR TI=(Cotrimoxazole) OR AB=(Cotrimoxazole) OR TI=("co-trimoxazole") OR AB=("co-trimoxazole") OR TI=(deprim) OR AB=(deprim) OR TI=(Drylin) OR AB=(Drylin) OR TI=(duratrimet) OR AB=(duratrimet) OR TI=(eltrianyl) OR AB=(eltrianyl) OR TI=(escoprim) OR AB=(escoprim) OR TI=(Eslectin) OR AB=(Eslectin) OR TI=(espectrin) OR AB=(espectrin) OR TI=(Eusaprim) OR AB=(Eusaprim) OR TI=(fectrim) OR AB=(fectrim) OR TI=(groprim) OR AB=(groprim) OR TI=(helveprim) OR AB=(helveprim) OR TI=(imexim) OR AB=(imexim) OR TI=(Kepinol) OR AB=(Kepinol) OR TI=(lagatrim) OR AB=(lagatrim) OR TI=(Lescot) OR AB=(Lescot) OR TI=(linaris) OR AB=(linaris) OR TI=(microtrim) OR AB=(microtrim) OR TI=(nopil) OR AB=(nopil) OR TI=(oecotrim) OR AB=(oecotrim) OR TI=(omsat) OR AB=(omsat) OR TI=(Oriprim) OR AB=(Oriprim) OR TI=(potesept) OR AB=(potesept) OR TI=(resprim) OR AB=(resprim) OR TI=("ro 6 2580-11") OR AB=("ro 6 2580-11") OR TI=("ro 62580") OR AB=("ro 62580") OR TI=(Septra) OR AB=(Septra) OR TI=(septran) OR AB=(septran) OR TI=(septrim) OR AB=(septrim) OR TI=(Septrin) OR AB=(Septrin) OR TI=(septrine) OR AB=(septrine) OR TI=(sigaprim) OR AB=(sigaprim) OR TI=(soltrim) OR AB=(soltrim) OR TI=(sulfamethoprim) OR AB=(sulfamethoprim) OR TI=("sulfamethoprim-ds") OR AB=("sulfamethoprim-ds") OR TI=("sulfamethoxazole and trimethoprim") OR AB=("sulfamethoxazole and trimethoprim") OR TI=("sulfamethoxazole plus trimethoprim") OR AB=("sulfamethoxazole plus trimethoprim") OR TI=("sulfamethoxazole trimethoprim") OR AB=("sulfamethoxazole trimethoprim") OR TI=(sulfaprim) OR AB=(sulfaprim) OR TI=(sulfatrim) OR AB=(sulfatrim) OR TI=("sulfatrim-ds") OR AB=("sulfatrim-ds") OR TI=("sulfatrim-ss") OR AB=("sulfatrim-ss") OR TI=(sulfotrim) OR AB=(sulfotrim) OR TI=(sulmeprim) OR AB=(sulmeprim) OR TI=(Sulprim) OR AB=(Sulprim) OR TI=(sumetrolim) OR AB=(sumetrolim) OR TI=(sumetrolin) OR AB=(sumetrolin) OR TI=(supracombin) OR AB=(supracombin) OR TI=(thiocuran) OR AB=(thiocuran) OR TI=("TMP SMX") OR AB=("TMP SMX") OR TI=("tms forte") OR AB=("tms forte") OR TI=(trib) OR AB=(trib) OR TI=(trigonyl) OR AB=(trigonyl) OR TI=(Trimedin) OR AB=(Trimedin) OR TI=("trimeth/sulfa") OR AB=("trimeth/sulfa") OR TI=("trimethoprim plus sulfamethoxazole") OR AB=("trimethoprim plus sulfamethoxazole") OR TI=("Trimethoprim Sulfamethoxazole") OR AB=("Trimethoprim Sulfamethoxazole") OR TI=(Trimethoprimsulfa) OR AB=(Trimethoprimsulfa) OR TI=(trimethoprimsulfamethoxazole) OR AB=(trimethoprimsulfamethoxazole) OR TI=("trimethoprim-sulfamethoxazole") OR AB=("trimethoprim-sulfamethoxazole") OR TI=("trimetoprim-sulfa") OR AB=("trimetoprim-sulfa") OR TI=(trimetoprimsulfamethoxazole) OR AB=(trimetoprimsulfamethoxazole) OR TI=(trimezol) OR AB=(trimezol) OR TI=(trimforte) OR AB=(trimforte) OR TI=(Trimosulfa) OR AB=(Trimosulfa) OR TI=(trimoxazole) OR AB=(trimoxazole) OR TI=("uro ts d") OR AB=("uro ts d") OR TI=("uroplus ds") OR AB=("uroplus ds") OR TI=("uroplus ss") OR AB=("uroplus ss")

**Semantic Scholar – limited to English language; 867 results; on 9.20.2024**

Medication AND (exposure OR intensity OR burden) AND acute kidney injury
